# Reprogramming the Aging Type H Vascular Niche via Myeloid Angiogenic Cell-Derived Extracellular Vesicles: Mechanisms, Engineering Strategies, and Translational Perspectives

**DOI:** 10.3390/ijms27188202

**Published:** 2026-09-15

**Authors:** Jinxin Zhang, Xingchen Wei, Na Han

**Affiliations:** 1 Key Laboratory of Trauma and Neural Regeneration, Ministry of Education, Peking University, Beijing 100044, China; zjx4244@gmail.com (J.Z.); cep0314@163.com (X.W.); 2 Department of Trauma and Orthopedics, Peking University People’s Hospital, Beijing 100044, China; 3 National Center for Trauma Medicine, Beijing 100044, China; 4 Department of Central Laboratory and Institute of Clinical Molecular Biology, Peking University People’s Hospital, Beijing 100044, China

**Keywords:** age-related osteoporosis, angiogenic-osteogenic coupling, myeloid angiogenic cells, extracellular vesicles, type H vessels, ferroptosis, senescence-associated secretory phenotype

## Abstract

Age-related osteoporosis is characterized by the simultaneous loss of bone mass and the regression of CD31^hi^Emcn^hi^ (Type H) vessels, a specialized capillary subtype that functionally couples angiogenesis with osteogenesis. The endothelial progenitor cell (EPC) compartment, comprising myeloid angiogenic cells (MACs) and endothelial colony-forming cells (ECFCs), contributes to the maintenance of this vascular niche; however, its regenerative potential declines with age, partly as a consequence of cellular senescence and acquisition of the senescence-associated secretory phenotype (SASP). Given the clinical hurdles of conventional cell transplantation, therapeutic development has shifted toward cell-free strategies based on the MAC/ECFC secretome, particularly extracellular vesicles (EVs). In this review, we clarify the distinction between MACs and ECFCs to resolve historical conceptual ambiguities, and examine how their paracrine cues regulate angiogenic–osteogenic coupling. We then synthesize current evidence on endothelial EV cargo and discuss how aging remodels vesicle composition and function to drive vascular–bone uncoupling. Beyond existing reviews on EPC-EVs, Type H vessels, or EV-based bone regeneration, we add a protocol-driven re-annotation and explicitly distinguish bone-related studies methodologically compatible with MAC-derived EVs from adjacent evidence using other endothelial cell sources and mechanistic support from non-skeletal models. Finally, we compare bioengineering strategies, including preconditioning, precision cargo engineering, surface targeting, and scaffold/hydrogel delivery, aimed at correcting senescence-associated cargo liabilities and improving translational readiness.

## 1. Introduction

As the global population ages, age-related osteoporosis has emerged as a profound public health burden. Recent meta-analyses indicate that the global prevalence of osteoporosis is approximately 18%, rising to nearly 35% in older women [1,2]. Alongside this trend, the incidence of fragility fractures continues to rise, particularly in the wrist and vertebrae [3]. Moreover, the two-year refracture rate following an index fracture can reach 11.58%, underscoring the profound vulnerability of these patients. While antiresorptive agents remain the clinical standard of care, a substantial treatment gap persists [4]. Closing this gap requires moving beyond simply halting bone resorption; it demands a deeper understanding of the upstream cellular mechanisms governing bone homeostasis to develop actively regenerative strategies.

Effective bone formation is not driven by osteoblasts in isolation; rather, it relies on a tightly coordinated process termed angiogenic–osteogenic coupling [5]. Central to this coupling is a specialized capillary subtype, CD31^hi^Emcn^hi^ (Type H) vessels. These vessels are spatially juxtaposed to osteoprogenitors and can instruct osteogenesis through hypoxia-inducible factor 1α (HIF-1α) and Notch signaling [6,7]. A progressive decline in Type H vessel density is increasingly recognized as a hallmark of skeletal aging. This rarefaction has been proposed as a vascular-level contributor to age-related bone loss, a concept supported primarily by correlative human data and mechanistic studies in preclinical models [8]. Because Type H vessels require continuous replenishment and renewal, their maintenance relies heavily on the functional integrity of the endothelial progenitor cell compartment, here discussed as myeloid angiogenic cells (MACs) and endothelial colony-forming cells (ECFCs). Aging erodes this vascular reserve: MACs/ECFCs become reduced in number and exhibit impaired angiogenic functions, including diminished migration and tubulogenesis [9,10]. These deficits ultimately uncouple the vascular–bone axis. Although autologous MAC/ECFC transplantation has been proposed as a therapeutic approach, clinical translation remains constrained by multiple barriers, including low engraftment efficiency, poor post-transplant survival, and intrinsic functional decline of aged autologous cells, with compromised proliferation and homing capacity [11].

To overcome these limitations, therapeutic development has increasingly shifted toward cell-free approaches leveraging the MAC/ECFC secretome. Within the paracrine repertoire of MACs/ECFCs, extracellular vesicles (EVs) are widely regarded as key mediators; their bioactive cargo, including proteins, lipids, and noncoding RNAs, can partially recapitulate the regenerative effects of their parent cells [12,13]. Compared with cell transplantation, these nanoscale vesicles generally offer improved stability and lower immunogenicity [14]. While several reviews have summarized Type H vessels and EV-based bone regeneration, many EPC-EV studies still rely on heterogeneous EPC definitions. Here, we explicitly distinguish MACs versus ECFCs based on immunophenotype and culture protocol, retrospectively annotate the likely cellular source of EPC-EVs where the reported isolation, culture, and phenotypic information permits such attribution, while retaining unclassified EPC-EVs when lineage assignment remains uncertain, and separate direct MAC-EV evidence in bone from adjacent endothelial-EV data and transferable mechanisms from non-skeletal ischemia models. We then examine how aging reshapes EV cargo composition and show bioengineering strategies aimed at restoring therapeutic efficacy in age-related osteoporosis.

Literature selection. We conducted a structured literature search in PubMed and Web of Science up to July 2026. Main keywords included combinations of “myeloid angiogenic cell(s)”/”MAC”, “endothelial colony-forming cell(s)”/”ECFC”, “endothelial progenitor cell(s)”/”EPC”, “extracellular vesicle(s)”/”EV”/”small EV(s)”/”exosome(s)”, “Type H vessel(s)”/”CD31^hi^Emcn^hi^”, “osteoporosis”/”aging”/”bone regeneration”, “senescence”/”SASP”, and “ferroptosis”. Additional relevant studies were identified by screening reference lists of key articles and recent reviews.

## 2. MAC/ECFC Subtypes and the Angiogenic–Osteogenic Niche

For years, studies of so-called endothelial progenitor cells in the bone microenvironment have been hampered by inconsistent terminology and a lack of standardized isolation protocols. A rigorous appraisal of the therapeutic value of progenitor-derived secretomes therefore requires that the underlying cellular heterogeneity be made explicit. Broadly, two populations must be distinguished: one with the capacity to directly contribute to vascular structure, and another that primarily orchestrates angiogenesis through paracrine signaling.

### 2.1. Heterogeneity and Evolution of the “EPC” Concept

In 1997, Asahara and colleagues isolated CD34^+^/KDR^+^ cells from human peripheral blood and demonstrated their incorporation into ischemic sites, introducing the concept of postnatal vasculogenesis [15]. These cells were initially regarded as bona fide endothelial precursors. However, subsequent stringent immunophenotyping revealed that cells obtained using conventional short-term adhesion protocols (typically on fibronectin-coated surfaces) are highly heterogeneous and predominantly of hematopoietic origin [16]. Rehman and others further showed that these early outgrowth cells mainly express monocyte/macrophage markers (CD14, CD45, CD11b) and lack definitive endothelial colony-forming capacity as well as a stable endothelial phenotype [17,18]. Accordingly, this population was reclassified as myeloid angiogenic cells (MACs), historically referred to as “early EPCs.”

Although MACs have limited proliferative capacity and rarely form perfused vascular lumens in vivo, they remain clinically relevant as potent paracrine hubs. MACs secrete high levels of proangiogenic mediators (e.g., VEGF, IL-8, G-CSF), thereby recruiting and supporting local/resident endothelial cells to execute vascular repair [19,20]. This distinction is essential for interpreting prior work in bone regeneration: in many studies where an “EPC-derived secretome” was applied to bone repair, the active products were in fact most consistent with MAC-derived secreted factors.

### 2.2. Immunophenotypic Definition of ECFCs

Subsequently, Ingram, Yoder, and colleagues identified a cell population that more closely matches a true endothelial progenitor phenotype, termed endothelial colony-forming cells (ECFCs), also known as “late EPCs” [21,22]. Unlike MACs, ECFCs typically expand only after 2–3 weeks of culture on type I collagen and display a characteristic cobblestone morphology. Immunophenotypically, ECFCs are negative for hematopoietic markers (CD45^−^, CD14) and positive for endothelial markers (CD31, VEGFR2, CD146) [22,23]. Functionally, ECFCs exhibit higher telomerase activity and robust clonal proliferative potential. Their principal therapeutic value lies in their ability to physically incorporate into nascent vascular networks and form perfused neovessels [21].

This dichotomy provides a useful lens for osteoporosis-related studies: ECFCs are more likely to contribute structurally to the Type H vasculature required for osteogenesis, whereas MACs primarily play an instructive and supportive role. Recognizing these differences helps resolve mechanism-of-action ambiguities: cell-free approaches (EVs/secretome) largely leverage MAC signaling capacity, whereas cell transplantation strategies often depend on the structural vasculogenic potential of ECFCs [24,25]. To reduce conceptual confusion, a recent consensus statement recommended retiring the catch-all term “EPC” in favor of nomenclature anchored in immunophenotype and function (Table 1) [25]. Terminological precision is a prerequisite for rigorous translation.

Where feasible, we therefore avoid the nonspecific label “EPC-EVs”. In subsequent sections, we retrospectively annotate vesicle sources as MAC-EVs or ECFC-EVs based on a strict review of isolation protocols; when lineage attribution cannot be confidently established from the original report, we use unclassified EPC-EVs to flag methodological gaps. Based on this methodological audit, most previously reported “EPC-EVs” in osteoporosis and bone repair align, in substance, with MAC-EVs.

### 2.3. Functional Dichotomy Within the Osteogenic Niche

Early work on EPC biology largely emerged from the cardiovascular field [26], yet the MAC/ECFC dichotomy is equally informative for osteoporosis pathophysiology. While MACs are unlikely to generate perfused luminal structures, they function as efficient paracrine factories, releasing abundant proangiogenic factors and EVs. In addition, MACs are more readily obtainable from peripheral blood than the comparatively rare ECFCs, making them a practical source of secretome-based therapeutics for cell-free strategies [20]. By contrast, the physiological attrition of ECFCs during aging is mechanistically linked to the rarefaction of Type H vessels, which in turn provides a vascular basis for impaired angiogenic–osteogenic coupling and progressive bone loss [27]. Distinguishing these subtypes therefore clarifies the therapeutic roadmap: ECFCs may be better suited for structural vascular replacement, whereas MAC secretomes can be leveraged to reprogram signaling networks and more precisely rejuvenate the aged microenvironment. Accordingly, this review focuses primarily on the latter, namely, the potential of MAC-derived paracrine signaling to restore the osteogenic niche.

## 3. Soluble Factors in the MAC Secretome That Regulate Bone Remodeling

**Figure 1:**
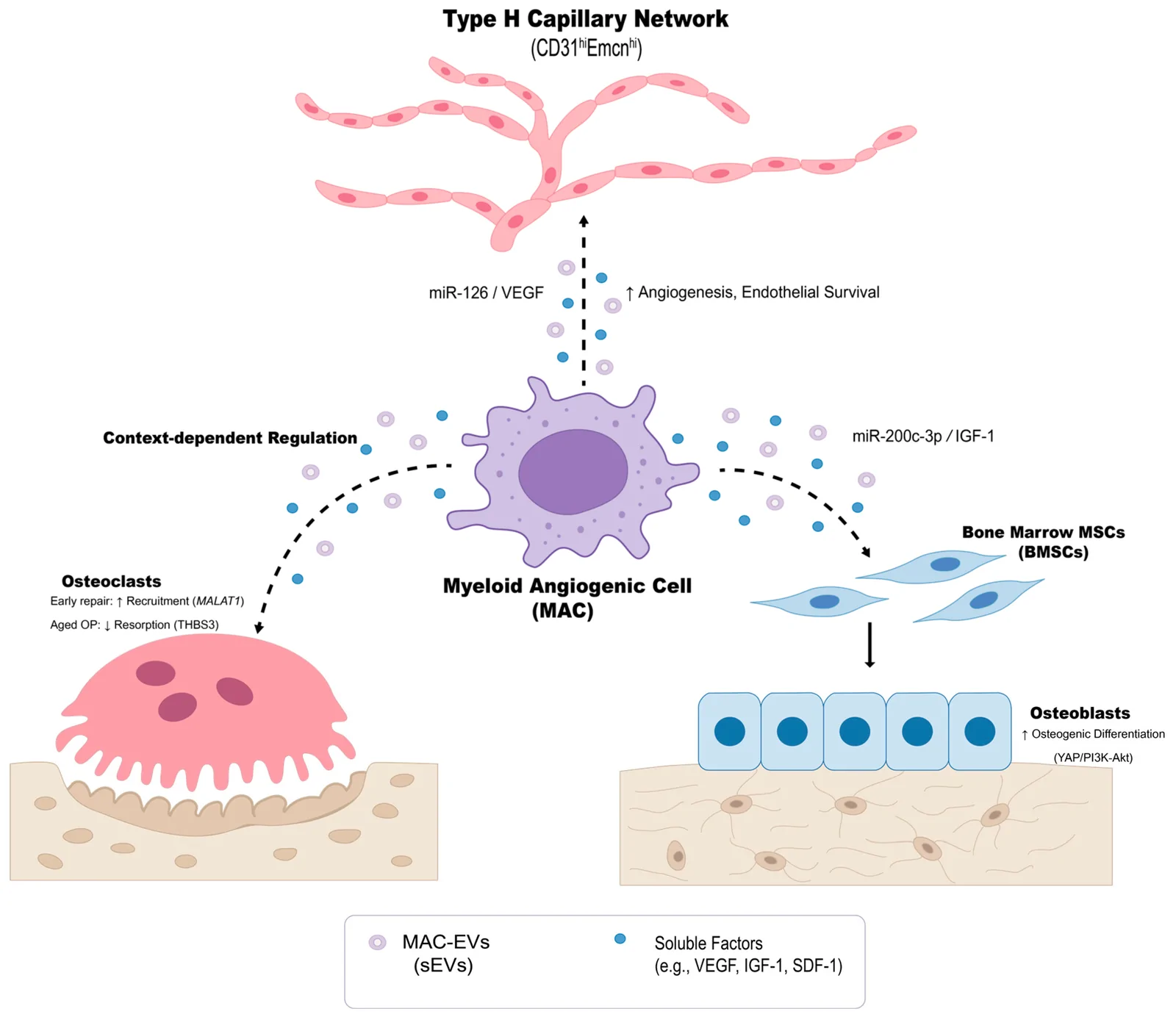
The paracrine regulatory network of MAC secretome within the angio-osteogenic niche. ↑ indicates upregulation/enhancement; ↓ indicates downregulation/inhibition.

Early therapeutic paradigms often emphasized vascular structural repair via transplantation of endothelial progenitor cells. Increasing evidence, however, indicates that the regenerative effects of these cells depend less on durable cellular incorporation and more on their paracrine outputs. In this section, we focus on soluble factors in the MAC secretome. EV-mediated signaling is discussed separately in Section 5. MACs function as metabolic and signaling hubs, releasing a complex mixture of soluble microenvironmental components, collectively termed the secretome, that coordinate angiogenic–osteogenic coupling [28]. Proteomics further suggests that this secretome is not a simple additive blend of growth factors (e.g., VEGF, IGF-1, PDGF, BMP-2) and chemokines (e.g., SDF-1α, IL-8); instead, it constitutes a dynamic signaling network shaped by matrix remodeling enzymes, particularly matrix metalloproteinases (MMPs) [29]. Acting in concert, these factors tune the bone marrow microenvironment and establish bidirectional communication among endothelial cells, bone marrow mesenchymal stromal cells (BMSCs), and osteoclasts (Figure 1).

### 3.1. Direct Pro-Osteogenic Regulation of BMSCs

Beyond promoting angiogenesis, MACs can directly enhance the anabolic program of BMSCs, thereby linking vascular cues to bone formation. VEGF is a central mediator of this process. In addition to its canonical proangiogenic activity, VEGF can act directly on BMSCs. Under conditions modeling diabetic osteoporosis, Wu and colleagues showed that MAC-derived VEGF binds VEGFR2 on BMSCs. This interaction drives F-actin–dependent cytoskeletal remodeling and promotes YAP nuclear translocation. As a mechanotransduction effector, nuclear YAP activates transcription of osteogenic genes such as Runx2 and Ocn(osteocalcin), enabling osteogenic differentiation even under metabolically compromised conditions [30,31].

The efficacy of the secretome also depends on tight control of factor bioavailability. IGF-1 represents another key MAC-secreted component. Han and colleagues reported that activation of PPAR-δ in MACs induces secretion of MMP-9, which cleaves IGFBP-3 and thereby liberates bioactive IGF-1. Once released, IGF-1 engages the PI3K/AKT/mTOR axis in recipient cells, a survival program that is particularly important for limiting apoptosis within the hypoxic fracture milieu [32,33]. The complexity of the secretome is further indicated by emerging regulators such as Serpine-1, which may enhance BMSC differentiation through a miR-181a-5p–dependent mechanism [29]. Regenerative outcomes appear to depend more on the stoichiometric balance among secretome components than on the presence or absence of any single factor. Song and colleagues described a “dose-dependent paradox”: when the VEGF/BMP-2 ratio becomes abnormally elevated, excessive VEGF induces Id1 upregulation in osteoblasts, which in turn impedes terminal differentiation by sequestering Runx2. Conversely, MAC-derived BMP-2 can directly initiate BMSC osteogenic differentiation via canonical Smad signaling, yet its efficacy is strongly constrained by concentration ratios with VEGF and other co-factors [34]. Together, these findings indicate that MAC secretomes must maintain a precise compositional balance to couple vascular invasion with bone mineralization.

### 3.2. Maintenance of Type H Vessels

As a structural prerequisite for osteogenesis, maintenance of Type H vessels depends on autocrine and paracrine feedback circuits initiated by MACs. SDF-1α/CXCL12 is one key mediator. Using a MAC cell-sheet model, Xue and colleagues demonstrated that MAC-secreted SDF-1α activates the CXCR4/PI3K/AKT/eNOS axis in an autocrine manner, enhancing MAC migration and homing while amplifying their proangiogenic activity [35].

Mechanical and environmental cues can also markedly reprogram the MAC secretome. In simulated microgravity, mechanical unloading suppresses MAC function at early stages. However, priming interventions can restore the secretory profile and increase NO, VEGF, and Ang-2 levels. These factors rescue endothelial function through HIF-1α/eNOS and downstream FAK/ERK1/2 signaling, enabling vascular ingrowth into fracture sites even under adverse biomechanical conditions [36,37]. In inflammatory microenvironments, MAC-derived PDGF-BB promotes a proangiogenic endothelial phenotype via PDGFRβ–Src–Akt signaling and can simultaneously recruit BMSCs to perivascular regions, thereby facilitating angiogenic–osteogenic coupling at a spatial level [38,39,40].

### 3.3. Time-Dependent Regulation of Osteoclastogenesis

The contribution of MACs to bone resorption is strongly context- and time-dependent. While preserving bone mass typically requires restraining osteoclast activity, MAC secretomes can, through multiple convergent pathways, enhance osteoclast precursor activation, an ostensibly dual effect that may be required during early phases of bone repair. For example, MAC-derived TGF-β1 upregulates Talin-1 to enhance adhesion and migration of bone marrow–derived macrophages, and activates Smad2/3 as well as the MAPK/ERK cascade to drive terminal differentiation into mature osteoclasts [41,42]. Chemotactic factors such as IL-8 can further promote an angiogenesis-permissive milieu and accelerate vascular invasion together with the physical space created by osteoclast-mediated matrix resorption [43].

**Figure 2:**
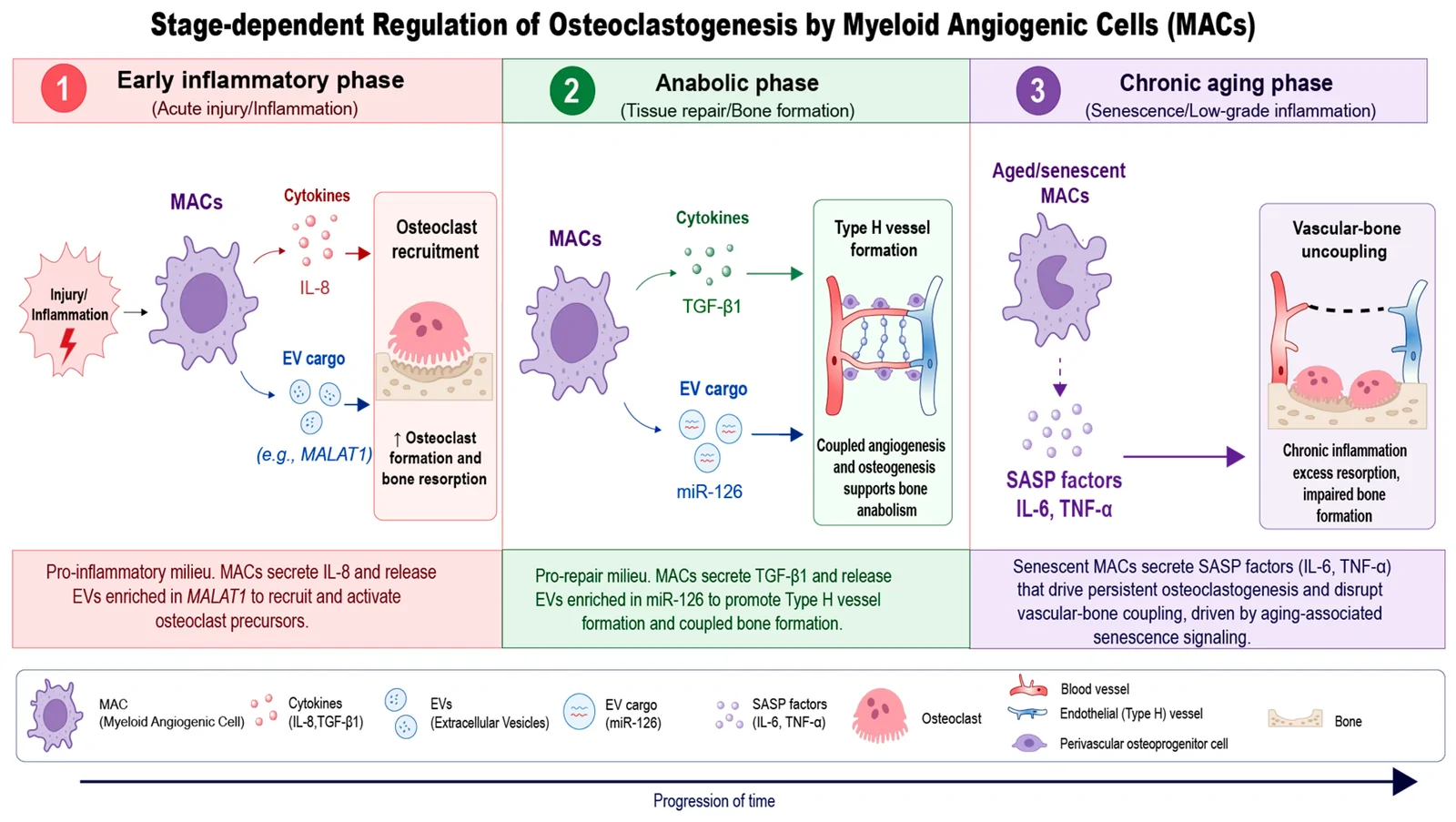
Stage-dependent regulation of osteoclastogenesis and coupling by MACs.

These features illustrate a broader principle: efficient bone formation often requires prior removal of damaged matrix. In acute fracture healing, such recruitment and activation can be beneficial. In age-related osteoporosis, however, the resorptive phase is frequently persistent and excessive; secretomes from senescent MACs, enriched in inflammatory mediators (e.g., IL-6, TNF-α) as part of the SASP, may exacerbate bone loss. Thus, MACs act as regulators of bone turnover, maintaining a dynamic balance between resorption and formation. Their control of osteoclastogenesis appears to be governed by a narrow temporal window, initiating remodeling early during repair, yet requiring timely shutdown during homeostasis. Age-related loss of this fine-tuning may contribute to the characteristic uncoupling observed in osteoporosis. To conceptualize this dynamic transition, we propose a stage-dependent model in which MAC-derived cytokines and EV cargo exert distinct regulatory roles across early inflammatory, anabolic, and chronic aging phases (Figure 2).

As illustrated in Figure 2, this stage-dependent model has direct therapeutic implications: interventions targeting the early inflammatory phase should aim to permit transient osteoclast-mediated matrix remodeling, whereas strategies for the anabolic and chronic aging phases should prioritize restoration of pro-osteogenic EV cargo and suppression of persistent SASP signaling. The molecular mechanisms underlying senescence-associated secretome reprogramming in MACs are examined in detail in Section 4.

## 4. Impaired Paracrine Function of MACs in Aging and Osteoporosis

While Section 3 focused on the temporal and niche-level consequences of MAC signaling (Figure 2), the following sections synthesize the intracellular molecular circuitry that drives aging-associated dysfunction. The therapeutic efficacy of MAC-EVs is functionally dictated by the pathophysiological state of the donor cells, which undergoes drastic remodeling during skeletal aging. In age-related osteoporosis, MACs decline in abundance, exhibit pronounced functional deterioration, and enter a senescent state [44]. This dysfunction is not limited to cell-cycle arrest. It also involves acquisition of SASP, which can fundamentally reprogram the bone microenvironment. Accordingly, effective rejuvenation strategies require a systematic understanding of how aging remodels the MAC secretome and how this, in turn, degrades the regenerative molecular cargo carried by EVs.

### 4.1. Molecular Drivers of the Senescent Phenotype

**Figure 3:**
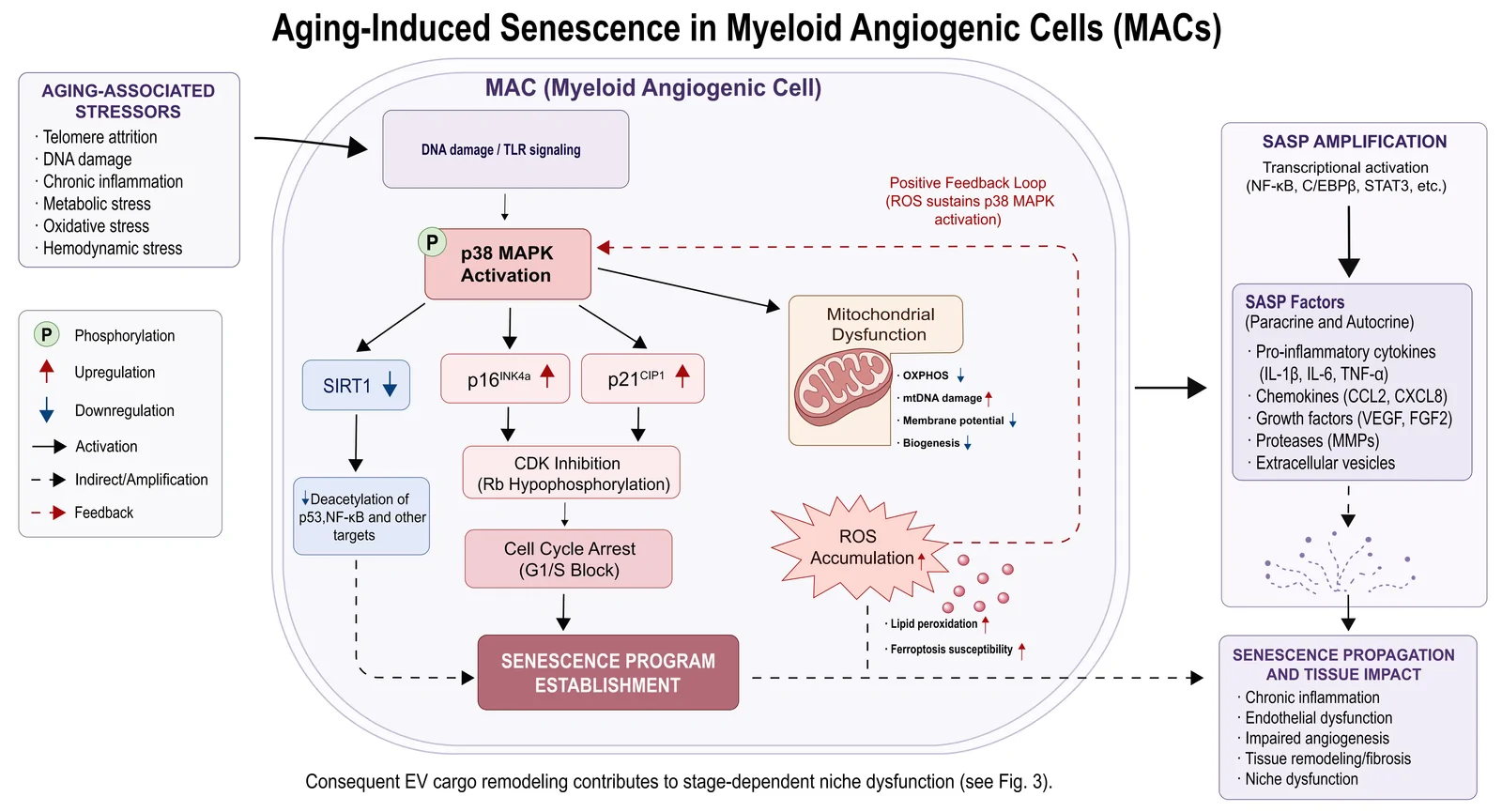
Aging-associated molecular stressors and the self-amplifying senescence network in MACs.

MAC senescence is driven by multiple convergent stressors, including telomere attrition, cumulative DNA damage, and metabolic stress. Under aging-associated chronic hyperglycemia or inflammatory conditions, excessive activation of the p38 mitogen-activated protein kinase (p38MAPK) pathway promotes cell-cycle arrest by upregulating inhibitors such as p16^INK4a^ and p21 [45,46]. This proliferative blockade is further compounded by epigenetic downregulation of SIRT1, thereby intensifying the senescent phenotype. These converging pathways form an integrated senescence signaling network rather than isolated linear events. The mechanistic interplay among p38 MAPK activation, SIRT1 downregulation, mitochondrial dysfunction, and ROS amplification is summarized in Figure 3.

As depicted in Figure 3, these interconnected pathways do not operate independently; rather, they form a self-amplifying network in which initial stressor-driven p38 MAPK activation progressively entraps MACs in a stable senescent state. This network also directly rewires the EV biogenesis machinery, altering cargo sorting programs in a manner that is examined in detail in Section 4.2.

Mechanistic studies indicate that increased miR-34a or cathepsin-mediated proteolytic cleavage can suppress SIRT1, leading to hyperacetylation of FoxO1 and PGC-1α, disruption of mitochondrial quality control, and accumulation of reactive oxygen species (ROS)—changes that stabilize and reinforce senescence [47,48,49].

In parallel with growth arrest, MAC metabolism shifts toward a proinflammatory secretory program. Senescent MACs show reduced stimulus-responsive secretion (for example, G-CSF release decreases by ~45%) yet actively produce SASP components such as IL-6, IL-8, and TNF-α [50]. This transition effectively converts MACs from facilitators of tissue repair into proinflammatory effector cells, aggravating local tissue injury and suppressing osteogenesis.

### 4.2. Pathological Remodeling of EV Cargo

Cellular senescence can directly rewire EV cargo and propagate senescent traits to neighboring cells via bystander effects. A hallmark of the aged secretome is depletion of regenerative microRNAs, most notably a marked reduction in miR-126. Aging MACs exhibit substantially reduced intracellular expression of miR-126 [51], a decline that is expected to translate into depleted angiomiR cargo in their derived EVs. Consistent with this, rejuvenation of senescent MACs via MSC-derived EVs partially restores miR-126-associated angiogenic signaling [52]. Loss of this angiomiR results in insufficient repression of SPRED1 in recipient cells, maintaining the vascular microenvironment in a low-VEGF–signaling state and thereby limiting effective angiogenesis. Evidence for aging-associated decline in pro-survival support is stronger at the cellular phenotype level than at the EV cargo level in the current MAC literature. For example, aging has been associated with a pro-apoptotic MAC phenotype with reduced Akt/Bcl-2–linked survival signaling [53], which is expected to impair the regenerative competence of their secretome/EVs. However, direct proteomic validation that Bcl-2/Akt are consistently depleted in aged MAC-EVs remains limited and should be addressed in future bone-focused studies.

Moreover, aged EVs may be enriched for pathological mediators with pro-senescent signatures. In the context of MAC aging, upregulation of miR-10a and miR-22, which target HMGA2 and AKT3 respectively, can induce premature senescence in recipient cells and compromise self-renewal capacity [54,55]. miR-21 exhibits highly context-dependent functions. Within senescent MACs, it has been reported to contribute to progenitor exhaustion. However, as discussed in Section 7.1, hypoxia-enriched miR-21-5p in non-myeloid progenitor-derived EVs can exert pro-angiogenic effects through distinct target networks, underscoring that miR-21’s role in EV biology is inseparable from its cellular and microenvironmental context. These changes can extend to epigenetic imbalance. For instance, SIRT1 deficiency promotes the release of inflammatory EVs, contributing to vascular inflammation and thrombosis [56,57]. Such cargo transfer helps explain why autologous MAC-based interventions often underperform in older patients: the therapeutic vehicle itself is already deteriorated by the aging process. The age-associated shift in MAC-derived EV cargo composition and its functional implications for the vascular–bone niche are summarized in Table 2.

### 4.3. Microenvironmental Crosstalk and Niche Deterioration

MAC senescence does not occur in isolation. It is catalyzed and perpetuated by a deteriorating bone marrow microenvironment. This niche imposes stress on local progenitors through both biochemical and physical mechanisms. Biochemically, the aged niche often manifests as inflammaging, a chronic low-grade inflammatory state: elevated TNF-α and IL-1β, together with accumulated ROS, can cause direct DNA damage and trigger a p38 MAPK–driven senescence cascade [58].

Physically, aging is associated with reduced blood flow and diminished shear stress within Type H vessels. Because endothelial Notch signaling cues (e.g., Jagged1 and DLL4) are mechanosensitive, diminished mechanical stimulation further downregulates coupling signals required for osteogenesis [59]. In addition, aged MACs exhibit aberrant calcium signaling with reduced CXCR4 expression, blunting their responsiveness to injury cues and impairing homing to fracture sites even when they remain in the circulation [60]. In sum, aging compromises MAC-mediated repair through intracellular senescence cascades, pathological EV cargo transfer, and an ROS-enriched adverse niche. Breaking this vicious cycle is a prerequisite for effective osteoporosis therapy.

## 5. Therapeutic Potential of MAC-Derived Extracellular Vesicles

As discussed in Section 4, the aged microenvironment can drive pathological remodeling of endogenous MAC cargo, most notably depletion of miR-126 and collapse of SIRT1-dependent antioxidant defense. Senescence-associated depletion of miR-126 and impairment of SIRT1-dependent antioxidant signaling may reduce the angiogenic and cytoprotective competence of MAC-derived EVs, potentially increasing the susceptibility of recipient cells to lipid peroxidation and ferroptosis. Therefore, delivering sEVs generated from rejuvenated or engineered MACs may serve as a targeted molecular replacement strategy to compensate for pathological cargo loss. Importantly, the strength of evidence varies across the literature: in Section 5, Section 6 and Section 7 we explicitly distinguish direct MAC-EV/sEV evidence in bone models from adjacent endothelial-EV studies (non-MAC sources) and transferable concepts derived from non-skeletal ischemia models. Throughout this review, the term ‘EVs’ is used as an umbrella designation encompassing all extracellular vesicle subtypes; ‘sEVs’ specifically refers to small EVs (<200 nm) as recommended by MISEV2023 [61]. We avoid using ‘exosomes’ as a synonym for sEVs unless endosomal origin is experimentally supported; when original studies use ‘exosomes’ without such evidence, we describe them as sEVs (termed ‘exosomes’ in the original report).

Beyond terminological clarity, EVs offer a practical pharmacological advantage over soluble factors. Clinically, soluble growth factors are often limited by rapid enzymatic degradation and restricted diffusion within dense bone matrix. By contrast, the lipid bilayer of MAC-EVs protects their payload from extracellular nuclease-mediated degradation, thereby improving stability over distance and time and enabling effective reprogramming of recipient cells within the bone marrow microenvironment.

### 5.1. Cargo Landscape of MAC-EVs and angiomiR Networks

As summarized in Section 4.2, aging is associated with depletion of regenerative angiomiRs, particularly miR-126, and broader deterioration of progenitor-cell angiogenic function [62,63]. In bone-related EV studies, the relevant functional readouts converge principally on endothelial survival and angiogenic signaling, including SPRED1–RAF/ERK and PI3K/Akt survival pathways. Beyond this canonical function, EV cargo exhibits marked plasticity in response to environmental cues and can be further augmented through genetic engineering to enrich therapeutic payloads. Under inflammatory or hypoxic conditions, MACs can reconfigure sorting programs to generate protective EV responses. For example, miR-126a-5p becomes enriched in MAC-EVs and can target the 3′-UTRs of inflammatory-factor mRNAs, thereby reducing TNF-α and IL-6 expression in brain microvascular endothelial cells in a non-skeletal model [64]. In addition, MAC-EVs can deliver miR-199a-3p and miR-30e-5p to suppress SP1, activate AMPK, and upregulate GPX4, forming a cytoprotective axis that lowers ferroptosis risk and preserves vascular integrity [65,66]. Although these anti-ferroptotic and anti-inflammatory mechanisms have been established mainly in cardiovascular and ischemia models, their relevance to the bone marrow microenvironment, characterized by lipid-rich adipocytes and altered iron metabolism during aging, remains to be systematically validated in skeletal contexts.

### 5.2. Rebuilding the Angiogenic–Osteogenic Niche

The Type H vascular niche structurally couples angiogenesis to osteogenesis. Its decline is a defining feature of osteoporosis, making restoration of Type H vessels a key therapeutic objective. MAC-EVs may support restoration of this niche through complementary angiogenic and osteogenic signaling mechanisms. In a distraction osteogenesis model, sEVs derived from bone marrow MACs, which are naturally enriched in miR-126, promoted capillary invasion and accelerated bone consolidation by inhibiting SPRED1 and thereby relieving repression of the Raf/ERK pathway [13]. Physical stimulation can also modulate EV cargo composition in osteogenic contexts. For instance, magnetic nanoparticle stimulation of BMSCs enriches BMSC-derived sEVs (termed ‘exosomes’ in the original report) in miR-1260a, which concurrently targets endothelial COL4A2 and HDAC7, enabling multi-target synergy between vascular and osteogenic programs [67]. Although this finding originates from BMSC-derived EVs rather than MACs, it illustrates the broader principle that physical preconditioning can synchronize vascular growth with bone matrix deposition, a strategy applicable to MAC-EV engineering.

In glucocorticoid-induced osteoporosis, MAC-EVs activate the PI3K/Akt/Bcl-2 survival axis to suppress osteoblast apoptosis and reduce femoral head necrosis, while upregulating System Xc- and GPX4 to block ferroptosis and protect osteoblasts [68,69]. Furthermore, in mature endothelial cell–derived sEVs (not MAC-EVs), pharmacologic priming (e.g., curcumin) has been reported to enrich sEV-associated miRNAs (e.g., miR-3p-975_4191) that synergistically suppress BMSC adipogenesis and bone marrow fat accumulation under osteoporotic conditions [70]. Intercellular interactions may also generate feed-forward amplification. In endothelial cell-derived EVs (cell subtype not specified as MAC/ECFC in the original report), vesicular miR-200c-3p was reported to regulate ZBTB16 and promote osteogenic differentiation of BMSCs; differentiated BMSCs subsequently secrete SLIT3, which enhances proliferation of Type H endothelium, thereby forming an angiogenic–osteogenic coupling loop [71]. Nonetheless, a translational gap remains (representative studies are summarized in Table 3). Most evidence is derived from acute injury or ovariectomy (OVX) models, which incompletely recapitulate the chronic ROS accumulation and lipid peroxidation characteristic of naturally aged age-related osteoporosis. Extrapolating efficacy from acutely inflamed fracture callus to an aged, adipose-enriched bone marrow niche should therefore be approached with caution. Future work should prioritize systematic validation in naturally aged mouse models. Notably, restoration of Type H vessels by engineered MAC-sEVs remains preclinical, with evidence to date largely limited to animal studies and without confirmed clinical efficacy in humans.

In contrast to MAC-EVs, ECFC-derived EVs remain substantially understudied in the context of bone vascular repair. Given that ECFCs are the primary cell type capable of physically incorporating into Type H vascular networks, their derived vesicles may carry distinct structural and vasculogenic cues, such as matrix-organizing proteins and endothelial junction-stabilizing factors, that complement the paracrine signaling of MAC-EVs. Systematic characterization of ECFC-EV cargo in aged bone marrow models represents an important and currently unaddressed research gap.

### 5.3. Context-Dependent Regulation of Bone Resorption

Extending the time-window concept outlined in Section 3.3, this section focuses on how MAC-EVs differentially regulate osteoclast activity across distinct remodeling contexts. Because the therapeutic goal in age-related osteoporosis is primarily to curb excessive resorption, anti-osteoclast mechanisms are of particular interest. Proteomics suggests that sEVs enriched in THBS3 can function as antiresorptive cargo: by binding CD47 on osteoclast precursors, THBS3 inhibits their differentiation while simultaneously promoting BMSC osteogenesis, thereby reversing bone loss in OVX models [73]. The THBS3-carrying vesicles in this study were derived from mature microvascular endothelial cells (EC-EVs) rather than MACs. Their inclusion here is justified by mechanistic relevance to the broader goal of endothelial EV-mediated antiresorptive therapy, rather than as direct evidence for MAC-EV cargo. Microvascular EV–delivered USP13 can stabilize NRF2, strengthen antioxidant defenses, and suppress ferroptosis-associated osteoclast maturation; the protein MANF can also attenuate osteoclastogenesis by downregulating NF-κB signaling [74]. These findings should be interpreted as adjacent endothelial-EV mechanisms, and their presence/abundance in MAC-sEVs remains to be established.

In acute fracture repair, by contrast, a transient increase in osteoclast activity is necessary to remodel mineralized cartilage callus. MAC-EVs carrying lncRNA-MALAT1 can sponge miR-124 to upregulate ITGB1 and promote osteoclastogenesis, accelerating the transition from soft to hard callus [72]. These findings underscore that clinical deployment of MAC-EVs will require precise temporal control: pro–bone-turnover cargo may be better suited for nonunion or early fracture repair, whereas antiresorptive cargo is more appropriate for systemic bone loss in age-related osteoporosis.

## 6. Translational Considerations and Standardization

Despite promising preclinical proof-of-concept, translating MAC-EVs into clinical orthopedics is severely bottlenecked by unresolved pharmaceutical and regulatory challenges. These challenges are both technical and systemic, centering on two priorities: engineering delivery systems that mitigate rapid in vivo clearance, and establishing scalable, regulator-ready manufacturing workflows to ensure product consistency and safety.

### 6.1. Systemic Delivery and Bone-Targeted Engineering

A central physiological barrier to cell-free EV therapy is the rapid systemic clearance of vesicles following administration. Native EVs exhibit short circulation half-lives and are predominantly sequestered by the mononuclear phagocyte system (MPS), particularly in the liver and spleen [75,76,77]. In addition, diffusion-driven dispersion and vascular washout from skeletal defects substantially reduce local bioavailability [78]. As a result, therapeutic efficacy is determined not only by EV cargo composition but also by spatial pharmacokinetics within regenerative microenvironments.

Most published hydrogel/scaffold delivery examples in bone regeneration to date use MSC- or BMSC-derived EVs rather than MAC-sEVs; we discuss them here as platform-level solutions that are likely transferable, while MAC-sEV–specific validation and release specifications remain necessary. To mitigate this “washout effect” and sustain therapeutically effective concentrations at target sites, recent bioengineering strategies have increasingly focused on biomimetic retention systems. Functionalized hydrogels that emulate key extracellular matrix (ECM) features provide structural confinement, prolong EV residence time, and modulate local immune and angiogenic signaling. Injectable hyaluronic acid–based matrices and self-healing peptide networks have emerged as preferred delivery vehicles for non–load-bearing defects, as they both retain EVs and establish a pro-angiogenic microenvironment conducive to vascular ingrowth and osteogenesis [78]. Mechanistically, such matrices may enhance integrin engagement, growth factor retention, and stiffness-mediated mechanotransduction signaling, thereby amplifying angiocrine responses.

In diabetic bone-defect models, MSC-derived engineered sEVs delivered via hydrogels (e.g., SHP2-enriched vesicles) restore endoplasmic reticulum homeostasis and mitochondrial function, overcoming metabolic constraints that impair regeneration under hyperglycemic conditions [79,80]. Similarly, self-healing chitosan/silk fibroin networks conform to irregular defects and function as sustained-release depots that maintain local EV bioactivity, thereby enhancing osteogenesis [81]. Incorporation of bone-targeting peptides (e.g., DSS6) into smart GelMA (gelatin methacryloyl) hydrogels enables hydroxyapatite-anchored localization and, when combined with ROS-scavenging nanoparticles, reshapes inflammatory microenvironments to favor regeneration [82]. Furthermore, modulation of photocrosslinked network stiffness allows fine-tuning of EV release kinetics, indirectly regulating macrophage polarization toward a pro-regenerative M2 phenotype [83].

For critical-sized segmental defects requiring structural load-bearing capacity, hydrogel systems alone are insufficient. In such contexts, 3D-printed scaffolds provide essential mechanical support while serving as EV reservoirs. Dopamine-coated β-TCP (beta-tricalcium phosphate) has been used to immobilize non-MAC EV preparations (e.g., sEVs termed ‘baicalin exosomes’ in the original study) and accelerate bone repair through suppression of inflammatory PRRX2/IL-6/AKT signaling [84]. This supports scaffold-based retention as a transferable delivery concept; MAC-sEV–specific loading, stability, and potency preservation require direct testing. Composite strategies integrating EVs with PEG/HA hydrogels and Bio-Oss improve particulate cohesiveness and enhance osteoinductive performance [85]. Functionalization of established orthopedic biomaterials with MAC-EVs thus represents a translationally feasible approach to augment graft bioactivity.

However, while biomimetic hydrogels and scaffold-based systems are highly effective for focal defects, age-related osteoporosis is inherently systemic and affects the entire skeletal architecture. Local implantation strategies cannot address diffuse trabecular loss [75]. Intravenous administration therefore appears attractive, yet systemic EV delivery remains constrained by dominant hepatic and splenic sequestration. Even with repeated dosing, only a modest fraction of circulating vesicles accumulates within bone tissue.

To overcome this limitation, bone-targeted surface engineering of EVs has gained increasing attention. Using click chemistry or lipid insertion techniques, bone-affinitive ligands such as Asp8 peptides or CH6 aptamers can be conjugated to the EV membrane to promote preferential homing to hydroxyapatite-rich or bone marrow niches [86,87,88]. Preclinical studies suggest that such modifications enhance skeletal accumulation and reduce off-target deposition, thereby improving the bioavailability of angiocrine cargo at osteoporotic lesions. Nevertheless, targeting efficiency remains incomplete, and quantitative biodistribution analyses indicate that MPS uptake is not entirely circumvented. Long-term safety, immunogenicity, and optimal dosing regimens for systemic bone-targeted EV therapy remain to be rigorously defined.

Collectively, these approaches represent a conceptual shift from passive retention toward active spatial targeting. For MAC-sEVs specifically, these delivery solutions should be evaluated against two MAC-linked constraints: donor- and state-dependent inflammatory/procoagulant risk (particularly when MACs are obtained from older individuals with inflammaging), and the possibility that enhanced bone targeting may still not rescue potency if senescence-associated cargo deficits are not corrected upstream (Section 4.2 and Section 7). Recent translational perspectives have emphasized aligning targeting engineering with scalable manufacturing and clinically relevant safety testing (e.g., repeat-dose tolerability and immunogenicity) [89,90,91].

### 6.2. Standardization, Scale-Up, and Regulatory Compliance

Heterogeneity in EV isolation and characterization remains a central obstacle to reproducibility and regulatory approval [92]. Whether for local sustained release or systemic targeting, clinical translation requires stable EV product quality and robust batch-to-batch consistency. The International Society for Extracellular Vesicles (ISEV) guidelines, MISEV 2018 and MISEV 2023, have become the field’s de facto gold standard [61,93,94]. Adherence is not merely procedural but scientifically essential, enabling investigators to distinguish bona fide EV-mediated effects from artifacts driven by co-isolated soluble proteins or protein aggregates [89].

Rigorous reporting is commonly framed around the five-component framework, which includes assessment of transmembrane markers (e.g., CD9, CD63) and cytosolic EV-associated proteins (e.g., TSG101), together with verification that common non-EV contaminants, such as albumin and lipoproteins, are minimized or absent [61,93]. The EV-TRACK initiative further promotes transparency through the EV-METRIC scoring system [95]. Moving toward clinical-scale production also demands a fundamental shift in isolation technology. Although ultracentrifugation (UC) remains common in basic research, it is labor-intensive, low-yield, and prone to inducing vesicle aggregation, limiting its suitability for manufacturing. Tangential flow filtration (TFF) is widely viewed as a superior alternative and can substantially improve purity in a single step (with albumin removal reported to be ~40-fold higher than UC) [96]. More recently, combining TFF with size-exclusion chromatography (SEC) has further improved purity and structural integrity while enabling processing of large volumes of conditioned media [97].

Clinical implementation must also comply with good manufacturing practice (GMP). Closed, sterile production workflows based on TFF have recently been applied to progenitor-derived secretome manufacturing and incorporate quality-control (QC) panels that include sterility, endotoxin testing (specification set according to the intended route/dose and pharmacopeial limits; e.g., <5 EU/mL reported in a GMP sEV product, often labeled as an ‘exosome’ product in the field), mycoplasma screening, and potency assays based on target-cell proliferation, establishing a foundation for Phase I studies [98,99]. Bioreactor platforms can support the production of clinical-grade sEV preparations, and lyophilization or cryopreservation in clinically approved buffers (e.g., Plasma-Lyte) can preserve EV bioactivity for months [100].

For MAC-sEV translation, standardization must extend beyond generic EV characterization to include donor qualification (age, metabolic/inflammatory status) and an explicit definition of the MAC source and activation state, because these variables can reshape SASP-linked cargo and safety-relevant attributes. Batch consistency should be monitored using predefined critical quality attributes (CQAs), including particle metrics, EV marker/contaminant panels, endotoxin/sterility, and inflammatory/procoagulant signatures where relevant [89].

Equally important are potency assays that reflect the intended mechanism in osteoporosis. Candidate potency assays for future validation could include endothelial functional readouts (migration/proliferation/tube formation), Type H relevant markers in bone microvascular endothelial cells (CD31^hi^Emcn^hi^ or a validated transcriptomic surrogate), and osteogenic outcomes in BMSCs (ALP activity, mineralization, and RUNX2/OSX/OCN expression). Given the growing emphasis on oxidative stress and ferroptosis in aged bone, optional mechanistic assays may include lipid peroxidation (e.g., C11-BODIPY) and GPX4/SLC7A11 rescue in recipient cells. Finally, biodistribution and repeat-dose safety (immunogenicity and thrombogenicity) should be incorporated early to de-risk systemic indications. Recent translational frameworks provide guidance on aligning CQAs, potency, and safety for EV therapeutics [90].

## 7. Future Directions: Enhancing EV Potency

**Figure 4:**
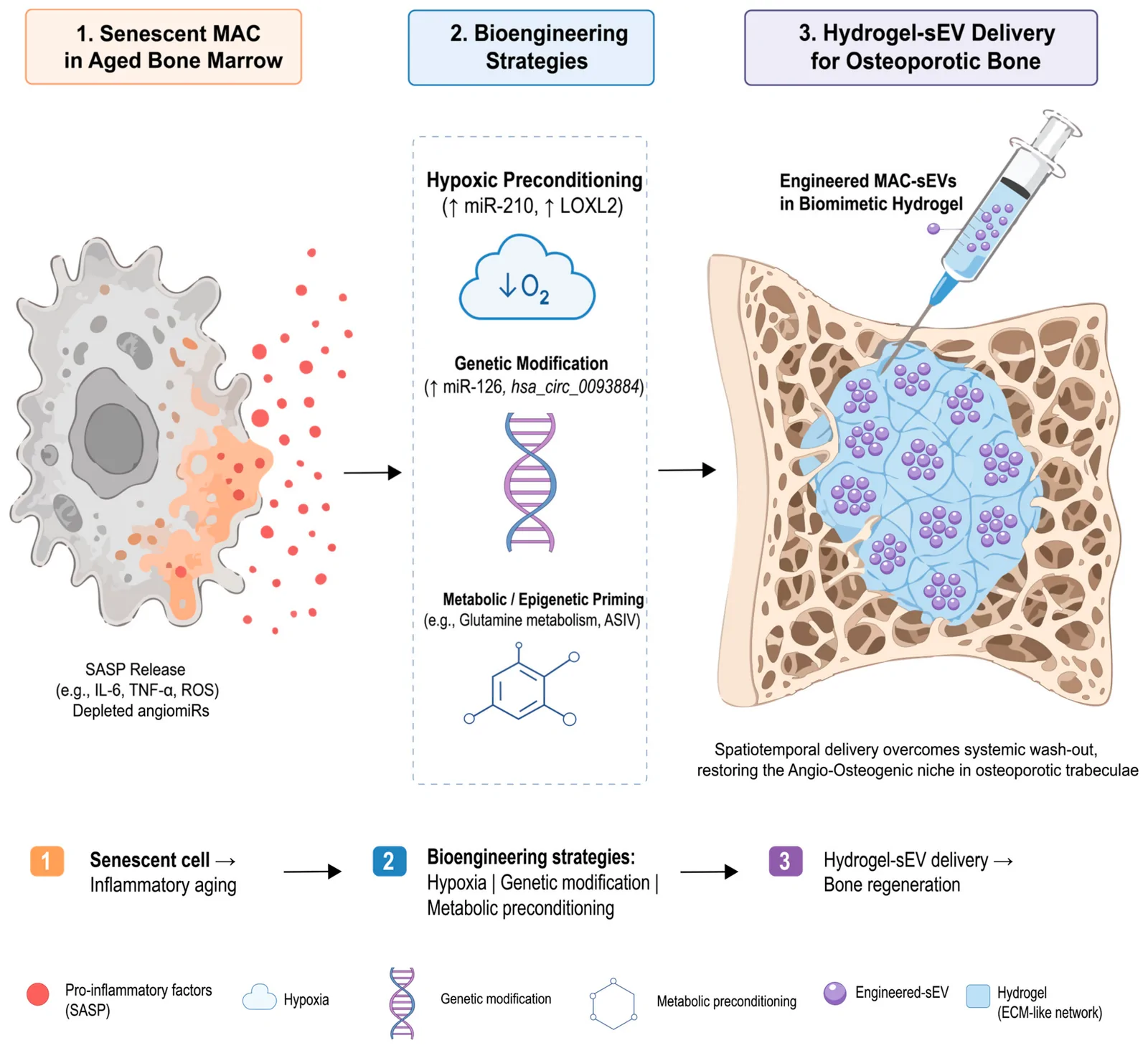
Translational roadmap for next-generation engineered MAC-sEVs to overcome senescence-associated bottlenecks. Black arrows indicate the sequential workflow/progression (from senescent cells to bioengineering strategies, and finally to hydrogel-sEV delivery). Upward arrows (↑) indicate upregulation or enrichment (e.g., ↑ miR-210, ↑ LOXL2). Downward arrow (↓) indicates reduction (e.g., ↓ O_2_ for hypoxia). → indicates a downstream effect, targeting, or causal relationship.

Section 6 addressed delivery and manufacturing bottlenecks—challenges that are partly agnostic to EV bioactivity. An equally critical upstream limitation is that MAC-sEV potency is constrained by donor heterogeneity and senescence-associated cargo deterioration, especially when vesicles are sourced from aged or diseased individuals. Importantly, the engineering literature relevant to osteoporosis is heterogeneous: many potency-enhancement studies are performed using non-MAC cell sources (e.g., MSCs or mature endothelial cells) or in non-skeletal ischemia models. Accordingly, in this section we organize engineering strategies as a comparative framework rather than a study-by-study list, and we explicitly indicate whether each example represents direct MAC-sEV evidence in bone or a transferable design principle requiring validation in aged bone [91]. We focus on four actionable layers, parent cell preconditioning, precision cargo engineering, surface targeting/immune-evasion, and scaffold/hydrogel-enabled delivery, and summarize their typical advantages and limitations for translation [101] (Figure 4).

### 7.1. Mimicking Hypoxia to Optimize Therapeutic Cargo

Physiological hypoxia is a key developmental cue for the vasculature. Accordingly, hypoxic conditioning (typically 1–5% O_2_) has emerged as an effective approach to boost EV bioactivity [102]. Hypoxic stress can selectively enrich proangiogenic microRNAs within vesicular cargo [102]. However, direct evidence that hypoxia consistently improves MAC-sEV potency in aged bone contexts remains limited. The following studies are presented primarily as transferable engineering principles unless otherwise specified. For example, although not derived from MACs, hypoxia-preconditioned adipose stem cell-derived sEVs (termed ‘exosomes’ in the original study) exhibit elevated miR-21-5p. When delivered via biomimetic hydrogels, these enhanced EVs can target SPRY1 and activate PI3K/AKT signaling, thereby accelerating Type H vessel formation and promoting repair of osteoporotic fractures [57]. It is important to note that this pro-angiogenic function of miR-21-5p is context-specific, operating in adipose-derived stromal cells under hypoxic preconditioning, and should be distinguished from the pro-senescent roles attributed to miR-21 in aged MACs (see Section 4.2). Hypoxic preconditioning can also reshape the proteomic landscape of the secretome. In hypoxia-preconditioned dental pulp stem cell (DPSC)-derived sEVs (non-MAC), vesicles were reported to show increased LOXL2, a factor critical for collagen crosslinking and vascular matrix stabilization [103]. Combining hypoxia with three-dimensional spheroid culture can produce synergistic gains in MSC systems: 3D architecture increases EV yield, while hypoxia promotes miR-210 loading, resulting in EVs with stronger antioxidant capacity that protect recipient cells from ROS-mediated apoptosis [104]. This hypoxia-associated signature may further act by delivering miR-210-3p to suppress TFR1-dependent ferroptosis, suggesting an additional mechanism for preserving vascular niches under oxidative stress in non-skeletal hypoxia-reoxygenation settings [105]. Its relevance to aged bone marrow endothelium remains to be validated. As an engineering strategy, preconditioning is attractive because it is scalable and avoids permanent genetic modification; however, it can introduce substantial batch variability and may induce unwanted inflammatory programs depending on the parent cell type and stimulus. For MAC-sEVs in osteoporosis, direct validation in aged bone models and predefined release criteria (e.g., pro-angiogenic potency without SASP-like inflammatory drift) are therefore essential.

### 7.2. Precision Cargo Engineering via Genetic Modification

Genetic engineering enables targeted enrichment of defined therapeutic molecules, thereby overcoming intrinsic limits of endogenous cargo sorting [106]. Transfection of parent cells using plasmids or viral vectors can increase the abundance of specific noncoding RNAs in EVs by orders of magnitude. Enriching circular RNAs is a promising strategy [107]. Overexpression of hsa_circ_0093884 in MACs promotes its loading into sEVs (termed ‘exosomes’ in the original report), where it functions as a miR-145 sponge. This decoy mechanism relieves repression of SIRT1, markedly enhancing neovascularization and wound-healing efficacy [108]. In mature endothelial cell–derived sEVs (not MAC-sEVs), vesicles were reported to package CRELD2 and activate the PERK–ATF4 arm of the unfolded protein response to promote osteogenesis and angiogenic–osteogenic coupling [109]. Electroporation can load VEGF plasmids into MAC-EVs to generate gene-activating sEVs (termed ‘exosomes’ in the original report) [110]. Compared with synthetic liposomes, these EVs show higher transfection efficiency and improved bone-regenerative outcomes, potentially leveraging endogenous EV homing while avoiding the immunogenicity of viral vectors [111].

Genetic strategies can also be deployed to strengthen anti-ferroptotic capacity. Enriching EVs with miR-30e-5p can target the SP1/AMPK axis and rescue endothelial cells from erastin-induced ferroptosis [65]. Likewise, EVs enriched in miR-137 can protect mitochondrial function and counter ferroptosis in an oxyhemoglobin-induced injury model by suppressing the COX2/PGE2 axis [112]. These findings suggest that genetic engineering can amplify endogenous protective programs that are otherwise insufficient in aged cells.

Compared with preconditioning, cargo engineering offers higher payload controllability and a clearer mechanism of action, but it also raises greater regulatory and safety scrutiny (e.g., vector-related risks, off-target effects, and product consistency across batches). For MAC-sEV translation, non-viral, transient, and quantifiable loading methods may be preferable when feasible, coupled with orthogonal cargo quantification and functional potency assays aligned to Type H–supportive angiogenesis and osteogenesis.

### 7.3. Pharmacologic and Epigenetic Priming

In clinical contexts where genetic manipulation may face regulatory barriers, priming MACs with small molecules or metabolites to reprogram the secretome offers a more readily translatable alternative [113]. Pharmacologic priming can increase EV output and potency: Astragaloside IV (ASIV) activates PI3K/AKT/mTOR signaling in MACs and markedly increases sEV release (often referred to as ‘exosome secretion’); ASIV-primed sEVs (termed ‘exosomes’ in the original report) exhibit enhanced proangiogenic activity and accelerate diabetic wound healing, accompanied by increased VEGF and CD31 expression [114]. Epigenetic regulation can also be harnessed for cargo remodeling. For instance, melatonin treatment of endothelial cells upregulates the m6A methyltransferase WTAP, promoting m6A-dependent maturation and EV loading of miR-302d-5p. These EVs can target Wnt3 in vascular smooth muscle cells to suppress vascular calcification [115]. These observations indicate that metabolic or epigenetic priming may correct aging-associated pathological cargo shifts and restore regenerative secretory programs without altering genomic sequences.

### 7.4. Surface Targeting and Immune Evasion

Beyond modulating cargo, engineering the EV membrane can improve systemic pharmacokinetics and skeletal tropism. For example, engineering EVs to display CD47 (‘don’t eat me’ signal) has been reported to reduce macrophage phagocytosis and prolong circulation time [116,117]. Bone-affinitive ligands (e.g., Asp-rich peptides or aptamers) and conjugation chemistries may increase bone accumulation, which is particularly relevant for systemic osteoporosis where local implantation is not feasible (see Section 6.1) [118,119]. The main limitations are incomplete escape from MPS sequestration, potential immunogenicity of added ligands, and added manufacturing complexity that can challenge batch consistency. For MAC-sEVs, membrane engineering should therefore be coupled to quantitative biodistribution readouts and repeat-dose safety testing to ensure that targeting gains translate into meaningful Type H–supportive activity in aged bone.

### 7.5. Scaffold/Hydrogel-Enabled Delivery

In contrast to systemic targeting, biomaterial-assisted delivery primarily addresses local bioavailability by mitigating washout and enabling sustained release. This approach is advantageous for focal defects and fracture sites, where retention can compensate for limited circulation time and may reduce the required dose [120]. However, it is less suited to diffuse systemic bone loss and introduces device-related regulatory considerations. When applied to MAC-sEVs, an important design goal is to preserve vesicle integrity and potency during immobilization/release while avoiding unintended inflammatory activation within the aged marrow niche [121].

In summary, across these engineering layers, hypoxia and 3D culture primarily enhance EV production and stress-adaptive cargo, whereas genetic/cargo engineering enables the most precise payload control. Pharmacologic and epigenetic priming sits between these extremes, while surface targeting and scaffold/hydrogel strategies address spatial delivery-systemic versus local, respectively.

## 8. Conclusions

Angiogenesis and osteogenesis cooperate to maintain skeletal integrity, with MACs and ECFCs acting as upstream regulators of this coupling. In age-related osteoporosis, activation of intrinsic aging programs together with an adverse inflammatory milieu drives functional decline across MAC/ECFC progenitor pools, thereby compromising the Type H vascular niche. Rarefaction of this vascular subtype represents a critical node that undermines the coupling between bone resorption and bone formation and accelerates the progression of skeletal fragility.

MAC-derived EVs offer a flexible cell-free therapeutic modality that may partially mitigate risks associated with whole-cell transplantation, including immune incompatibility and tumorigenicity. Importantly, these nanoscale vesicles are not inert carriers; they function as biologically instructive units. Available studies suggest that MAC-compatible EPC-EVs can deliver proangiogenic regulatory RNAs such as miR-126 and influence survival and ferroptosis-related pathways in recipient cells. Together with mechanistic evidence from other endothelial EV systems, these findings support the possibility that engineered MAC-EVs could promote angiogenesis, support osteogenesis, and modulate bone remodeling; however, this integrated therapeutic profile remains to be validated in naturally aged osteoporosis models. This pleiotropic profile contrasts with conventional antiresorptive drugs, which often suppress bone turnover in a largely nonselective manner, although this mechanistic advantage has thus far been demonstrated predominantly in rodent models and awaits validation in human clinical settings.

Translating these biological advantages into clinical therapies, however, requires overcoming substantial methodological, pharmacokinetic, and safety barriers. The field must move beyond heterogeneous isolation workflows toward scalable, MISEV-compliant manufacturing processes (e.g., TFF) to ensure reproducibility. Furthermore, the safety profile of MAC-derived EVs, particularly regarding potential pro-inflammatory cargo from senescent donor cells and the immunogenicity of genetically engineered preparations, must be rigorously characterized in dedicated preclinical safety studies before human trials can be initiated. Given the rapid in vivo clearance of native EVs, biomaterial-enabled spatiotemporal delivery, using functionalized hydrogels and 3D-printed scaffolds, remains a prerequisite for many local bone-repair indications. Moreover, simply administering autologous EVs from older individuals is unlikely to achieve robust efficacy. A central future direction is next-generation engineering, through hypoxic preconditioning, genetic modification, or epigenetic priming, to proactively correct aging-induced cargo deterioration and enrich regenerative payloads prior to administration.

Over-reliance on antiresorptive pharmacotherapy risks overlooking the foundational role of vascular niches in bone homeostasis. Targeting restoration of the CD31^hi^Emcn^hi^ vascular niche with engineered MAC-EVs represents a promising and testable therapeutic strategy: rather than merely slowing bone loss, this cell-free strategy aims to actively reawaken the aged skeletal microenvironment and provides a mechanistic blueprint for restoring structural and metabolic resilience in aging bone.

## Figures and Tables

**Table 1:** Characterization and Definitions of Endothelial Progenitor Cell Subtypes.

Feature	MACs (Early EPCs)	ECFCs (Late EPCs)
Origin	Hematopoietic lineage (Bone Marrow/Monocytes)	Endothelial lineage (Vascular Wall/Bone Marrow)
Culture Appearance	Spindle-shaped; appear within 3–7 days	Cobblestone-like; appear after 14–21 days
Key Surface Markers	CD45^+^, CD14^+^, CD11b^+^, CD133^+/−^, CD31^−/dim^; CD146^−^, Tie2^−^	CD31^+^, CD146^+^, VEGFR2 (KDR)^+^, CD105^+^; CD34^+/−^; CD45^−^, CD14^−^, CD133^−^
Proliferative Potential	Low; limited lifespan; senescence-prone	High; high telomerase activity; robust clonal expansion
Mechanism of Action	Paracrine signaling (VEGF, IL-8, MMP-9, EVs)	Vasculogenesis; physical incorporation into neovessels
Relevance to Bone	Immune modulation; BMSC recruitment; source of therapeutic EVs	Structural formation of Type H vessels; coupling with osteoprogenitors.
Secretory Profile	High paracrine activity; highly susceptible to SASP with aging.	Matrix-associated factors; lower cytokine volume; provide structural niche support.

**Table 2:** Age-dependent remodeling of MAC-derived extracellular vesicle cargo and functional consequences in the angio-osteogenic niche.

Category	Young MAC-EVs	Aged MAC-EVs	Functional Consequences in Bone Niche	Representative Mechanisms/Pathways
AngiomiRs	Enriched miR-126a	miR-126 depletion	Impaired EC proliferation, migration; ↓ Type H vessels	SPRED1 derepression → ↓ VEGFR2/RAF/ERK
Pro-survival/Anti-apoptotic	High SIRT1	Reduced SIRT1	↑ Susceptibility to apoptosis and oxidative stress	SIRT1-FoxO1 disruption; PI3K/Akt attenuation; Caspase activation
Anti-ferroptotic	Robust GPX4, redox balance	Antioxidant defense failure	Vascular instability; osteoblast/EC ferroptosis	System Xc^−^/GPX4 axis impairment
Inflammatory mediators (SASP)	Low IL-6, TNF-α	Enriched IL-6, TNF-α, miR-21/22	Propagation of inflammaging; premature senescence in recipients	NF-dB activation; targeting HMGA2/AKT3
Regulatory ncRNAs/Epigenetics	Balanced miRNA network	Dysregulated miRNAs; SIRT1 suppression	Impaired progenitor self-renewal; vascular-bone uncoupling	Chromatin remodeling imbalance
Matrix remodeling	Balanced MMPs and Growth factors	Dysregulated MMP-9; altered IGF-1 release	Disrupted coupling of angiogenesis and osteogenesis	MMP-9–IGF-1 axis dysregulation

Arrows: ↑ indicates upregulation/enhancement; ↓ indicates downregulation/inhibition; → indicates a downstream effect, targeting, or causal relationship (e.g., A→B means A regulates B).

**Table 3:** Summary of Representative Studies on EPC/EC-Derived EVs in Bone Regeneration and Osteoporosis.

Model/ Pathology *	Parental Cell/ Isolation Method	Subtype Re-Classification	Key Cargo	Target Niche/ Mechanism	Major Outcome	Translational Gaps/Limitations	Ref.
Distraction Osteogenesis (Rat)	Rat Bone Marrow EPCs; Ultracentrifugation (UC)	Methodologically aligns with MAC-EVs (Short-term adhesion)	miR-126	HUVECs: ↓ SPRED1 → ↑ Raf/ERK pathway	Accelerated capillary invasion and enhanced bone consolidation.	Used naive young cells; Local injection without controlled release system.	[13]
Femur Fracture (Mouse)	Mouse EPCs; UC	Unclassified EPC-EVs (Ambiguous lineage tracking)	LncRNA- MALAT1 miR-124 → ↑ Integrin β1	Osteoclasts: Sponging	Promoted osteoclast recruitment for early soft callus remodeling.	Potential risk in osteoporotic or aged settings where excessive osteoclast activation may impair net bone mass.	[72]
Aged and OVX OP (Mice)	Human Microvascular ECs; UC	EC-EVs (Mature Endothelial Cells)	THBS3 (Protein)	BMSCs: Binding CD47 receptor	Dual effect: ↑ Osteogenesis ↓ Adipogenesis.	Derived from mature ECs rather than progenitors; limited regenerative yield and replicative capacity.	[73]
Steroid- Induced OP (Mice)	Mouse Bone Marrow EPCs; Polymer-based precipitation kit	Methodologically aligns with MAC-EVs	Generic Cargo	Osteoblasts: ↑ System Xc-/GPX4 axis	Prevented femoral head necrosis; reduced osteoblast ferroptosis.	Used unmodified naive EVs without preconditioning; compliance with MISEV 2023 purity standards lacks detail.	[68]
OVX OP (Mice)	Human Microvascular ECs; UC	EC-EVs (Mature Endothelial Cells)	miR-3p-975_4191	BMSCs: ↓ TNF-α signaling	Synergistic promotion of osteogenesis and suppression of adipogenesis via TNF targeting.	Synergistic effect validated in vitro; in vivo combinational efficacy and safety remain unverified.	[70]
Stroke/Ischemia (Mouse) *	Mouse EPCs (Transfected with miR-126 mimic); UC	Methodologically aligns with MAC-EVs	miR-126	Local ECs: ↓ Caspase-3; ↑ VEGFR2	Reduced infarct volume; promoted robust angiogenesis.	Extrapolation gap: Investigated neuro-vascular niche; efficacy in restoring Type H bone capillaries requires validation.	[62]
Atherosclerosis (Mouse) *	Mouse Cord Blood EPCs; UC	ECFC-EVs (Late outgrowth morphology)	miR-199a-3p	Vascular ECs: ↓ SP1 → ↑ SLC7A11/GPX4 → ↓ ferroptosis	Protected endothelium from lipid peroxidation and ferroptosis.	Extrapolation gap: Focuses on large vessels rather than bone marrow microvasculature.	[66]

Note for Table 3: Arrows: ↑ indicates upregulation/enhancement; ↓ indicates downregulation/inhibition; → indicates a downstream effect, targeting, or causal relationship (e.g., A→B means A regulates B). Studies utilizing non-endothelial/progenitor lineages (e.g., BMSCs, macrophages) were excluded. For studies labeled as “EPCs” by the original authors, we reclassified the likely cellular source according to the criteria in Table 1. When isolation/culture or phenotypic information was insufficient for attribution, vesicles were designated as unclassified EPC-EVs. Asterisks (*) indicate proof-of-concept models outside bone, cited as transferable mechanistic support rather than direct MAC-EV evidence. Where original studies used "exosomes" without demonstrating endosomal origin, these are referred to as sEVs in accordance with MISEV2023.

## Data Availability

No new data were created or analyzed in this study. Data sharing is not applicable to this article.

## References

[1] Salari N., Ghasemi H., Mohammadi L., Behzadi M.H., Rabieenia E., Shohaimi S., Mohammadi M. (2021). The Global Prevalence of Osteoporosis in the World: A Comprehensive Systematic Review and Meta-Analysis. J. Orthop. Surg. Res..

[2] Salari N., Darvishi N., Bartina Y., Larti M., Kiaei A., Hemmati M., Shohaimi S., Mohammadi M. (2021). Global Prevalence of Osteoporosis among the World Older Adults: A Comprehensive Systematic Review and Meta-Analysis. J. Orthop. Surg. Res..

[3] Xu B., Radojčić M.R., Anderson D.B., Shi B., Yao L., Chen Y., Feng S., Lee J.H., Chen L. (2024). Trends in Prevalence of Fractures among Adults in the United States, 1999–2020: A Population-Based Study. Int. J. Surg..

[4] Wong R.M.Y., Wong P.Y., Liu C., Wong H.W., Chung Y.L., Chow S.K.H., Law S.W., Cheung W.H. (2022). The Imminent Risk of a Fracture-Existing Worldwide Data: A Systematic Review and Meta-Analysis. Osteoporos. Int..

[5] Maes C., Clemens T.L. (2014). Angiogenic-Osteogenic Coupling: The Endothelial Perspective. BoneKEy Rep..

[6] Kusumbe A.P., Ramasamy S.K., Adams R.H. (2014). Coupling of Angiogenesis and Osteogenesis by a Specific Vessel Subtype in Bone. Nature.

[7] Ramasamy S.K., Kusumbe A.P., Wang L., Adams R.H. (2014). Endothelial Notch Activity Promotes Angiogenesis and Osteogenesis in Bone. Nature.

[8] Wang L., Zhou F., Zhang P., Wang H., Qu Z., Jia P., Yao Z., Shen G., Li G., Zhao G. (2017). Human Type H Vessels Are a Sensitive Biomarker of Bone Mass. Cell Death Dis..

[9] Shi H., Zhao Z., Jiang W., Zhu P., Zhou N., Huang X. (2022). A Review into the Insights of the Role of Endothelial Progenitor Cells on Bone Biology. Front. Cell Dev. Biol..

[10] Keighron C., Lyons C.J., Creane M., O’Brien T., Liew A. (2018). Recent Advances in Endothelial Progenitor Cells toward Their Use in Clinical Translation. Front. Med..

[11] Holkar K., Vaidya A., Pethe P., Kale V., Ingavle G. (2020). Biomaterials and Extracellular Vesicles in Cell-Free Therapy for Bone Repair and Regeneration: Future Line of Treatment in Regenerative Medicine. Materialia.

[12] Xing Z., Zhao C., Liu H., Fan Y. (2020). Endothelial Progenitor Cell-Derived Extracellular Vesicles: A Novel Candidate for Regenerative Medicine and Disease Treatment. Adv. Healthc. Mater..

[13] Jia Y., Zhu Y., Qiu S., Xu J., Chai Y. (2019). Exosomes Secreted by Endothelial Progenitor Cells Accelerate Bone Regeneration during Distraction Osteogenesis by Stimulating Angiogenesis. Stem Cell Res. Ther..

[14] Terriaca S., Fiorelli E., Scioli M.G., Fabbri G., Storti G., Cervelli V., Orlandi A. (2021). Endothelial Progenitor Cell-Derived Extracellular Vesicles: Potential Therapeutic Application in Tissue Repair and Regeneration. Int. J. Mol. Sci..

[15] Asahara T., Murohara T., Sullivan A., Silver M., van der Zee R., Li T., Witzenbichler B., Schatteman G., Isner J.M. (1997). Isolation of Putative Progenitor Endothelial Cells for Angiogenesis. Science.

[16] Takahashi T., Kalka C., Masuda H., Chen D., Silver M., Kearney M., Magner M., Isner J.M., Asahara T. (1999). Ischemia- and Cytokine-Induced Mobilization of Bone Marrow-Derived Endothelial Progenitor Cells for Neovascularization. Nat. Med..

[17] Rehman J., Li J., Orschell C.M., March K.L. (2003). Peripheral Blood “Endothelial Progenitor Cells” Are Derived from Monocyte/Macrophages and Secrete Angiogenic Growth Factors. Circulation.

[18] Rohde E., Bartmann C., Schallmoser K., Reinisch A., Lanzer G., Linkesch W., Guelly C., Strunk D. (2007). Immune Cells Mimic the Morphology of Endothelial Progenitor Colonies in Vitro. Stem Cells.

[19] Hur J., Yoon C.-H., Kim H.-S., Choi J.-H., Kang H.-J., Hwang K.-K., Oh B.-H., Lee M.-M., Park Y.-B. (2004). Characterization of Two Types of Endothelial Progenitor Cells and Their Different Contributions to Neovasculogenesis. Arterioscler. Thromb. Vasc. Biol..

[20] Yoon C.-H., Hur J., Park K.-W., Kim J.-H., Lee C.-S., Oh I.-Y., Kim T.-Y., Cho H.-J., Kang H.-J., Chae I.-H. (2005). Synergistic Neovascularization by Mixed Transplantation of Early Endothelial Progenitor Cells and Late Outgrowth Endothelial Cells. Circulation.

[21] Ingram D.A., Mead L.E., Tanaka H., Meade V., Fenoglio A., Mortell K., Pollok K., Ferkowicz M.J., Gilley D., Yoder M.C. (2004). Identification of a Novel Hierarchy of Endothelial Progenitor Cells Using Human Peripheral and Umbilical Cord Blood. Blood.

[22] Yoder M.C., Mead L.E., Prater D., Krier T.R., Mroueh K.N., Li F., Krasich R., Temm C.J., Prchal J.T., Ingram D.A. (2007). Redefining Endothelial Progenitor Cells via Clonal Analysis and Hematopoietic Stem/Progenitor Cell Principals. Blood.

[23] Timmermans F., Van Hauwermeiren F., De Smedt M., Raedt R., Plasschaert F., De Buyzere M.L., Gillebert T.C., Plum J., Vandekerckhove B. (2007). Endothelial Outgrowth Cells Are Not Derived from CD133^+^ Cells or CD45^+^ Hematopoietic Precursors. Arterioscler. Thromb. Vasc. Biol..

[24] Kalka C., Masuda H., Takahashi T., Kalka-Moll W.M., Silver M., Kearney M., Li T., Isner J.M., Asahara T. (2000). Transplantation of Ex Vivo Expanded Endothelial Progenitor Cells for Therapeutic Neovascularization. Proc. Natl. Acad. Sci. USA.

[25] Medina R.J., Barber C.L., Sabatier F., Dignat-George F., Melero-Martin J.M., Khosrotehrani K., Ohneda O., Randi A.M., Chan J.K.Y., Yamaguchi T. (2017). Endothelial Progenitors: A Consensus Statement on Nomenclature. Stem Cells Transl. Med..

[26] Hill J.M., Zalos G., Halcox J.P.J., Schenke W.H., Waclawiw M.A., Quyyumi A.A., Finkel T. (2003). Circulating Endothelial Progenitor Cells, Vascular Function, and Cardiovascular Risk. N. Engl. J. Med..

[27] Mund J.A., Estes M.L., Yoder M.C., Ingram D.A., Case J. (2012). Flow Cytometric Identification and Functional Characterization of Immature and Mature Circulating Endothelial Cells. Arterioscler. Thromb. Vasc. Biol..

[28] Rafii S., Butler J.M., Ding B.-S. (2016). Angiocrine Functions of Organ-Specific Endothelial Cells. Nature.

[29] Asbi T., Tamari T., Doppelt-Flikshtain O., Regev-Yehishalom O., Zusmanovich I., Younis A., Ogen T., Berg T., Bianco-Peled H., Zigdon-Giladi H. (2026). Proteomic Analysis of Endothelial Progenitor Cells Secretome Identifies Serpine 1 as a Potent Regulator of Osteogenesis. Sci. Rep..

[30] Wu P., Zhang X., Hu Y., Liu D., Song J., Xu W., Tan H., Lu R., Zheng L. (2021). Co-Culture with Endothelial Progenitor Cells Promotes the Osteogenesis of Bone Mesenchymal Stem Cells via the VEGF-YAP Axis in High-Glucose Environments. Int. J. Med. Sci..

[31] Li R., Nauth A., Gandhi R., Syed K., Schemitsch E.H. (2014). BMP-2 mRNA Expression after Endothelial Progenitor Cell Therapy for Fracture Healing. J. Orthop. Trauma.

[32] Han J.-K., Kim H.-L., Jeon K.-H., Choi Y.-E., Lee H.-S., Kwon Y.-W., Jang J.-J., Cho H.-J., Kang H.-J., Oh B.-H. (2013). Peroxisome Proliferator-Activated Receptor-δ Activates Endothelial Progenitor Cells to Induce Angio-Myogenesis through Matrix Metallo-Proteinase-9-Mediated Insulin-like Growth Factor-1 Paracrine Networks. Eur. Heart J..

[33] Hynes B., Kumar A.H.S., O’Sullivan J., Klein Buneker C., Leblond A.-L., Weiss S., Schmeckpeper J., Martin K., Caplice N.M. (2013). Potent Endothelial Progenitor Cell-Conditioned Media-Related Anti-Apoptotic, Cardiotrophic, and pro-Angiogenic Effects Post-Myocardial Infarction Are Mediated by Insulin-like Growth Factor-1. Eur. Heart J..

[34] Song X., Liu S., Qu X., Hu Y., Zhang X., Wang T., Wei F. (2011). BMP2 and VEGF Promote Angiogenesis but Retard Terminal Differentiation of Osteoblasts in Bone Regeneration by Up-Regulating Id1. Acta Biochim. Biophys. Sin..

[35] Xue F., Bai Y., Jiang Y., Liu J., Jian K. (2022). Construction and a Preliminary Study of Paracrine Effect of Bone Marrow-Derived Endothelial Progenitor Cell Sheet. Cell Tissue Bank..

[36] Kong L., Wang Y., Wang H., Pan Q., Zuo R., Bai S., Zhang X., Lee W.Y., Kang Q., Li G. (2021). Conditioned Media from Endothelial Progenitor Cells Cultured in Simulated Microgravity Promote Angiogenesis and Bone Fracture Healing. Stem Cell Res. Ther..

[37] Akita T., Murohara T., Ikeda H., Sasaki K.-I., Shimada T., Egami K., Imaizumi T. (2003). Hypoxic Preconditioning Augments Efficacy of Human Endothelial Progenitor Cells for Therapeutic Neovascularization. Lab. Investig. J. Tech. Methods Pathol..

[38] Wyler von Ballmoos M., Yang Z., Völzmann J., Baumgartner I., Kalka C., Di Santo S. (2010). Endothelial Progenitor Cells Induce a Phenotype Shift in Differentiated Endothelial Cells towards PDGF/PDGFRβ Axis-Mediated Angiogenesis. PLoS ONE.

[39] He S., Hou T., Zhou J., Ai Q., Dou C., Luo F., Xu J., Xing J. (2022). Endothelial Cells Promote Migration of Mesenchymal Stem Cells via PDGF-BB/PDGFRβ-Src-Akt in the Context of Inflammatory Microenvironment upon Bone Defect. Stem Cells Int..

[40] Fang J., Huang X., Han X., Zheng Z., Hu C., Chen T., Yang X., Ouyang X., Chen Z., Wei H. (2020). Endothelial Progenitor Cells Promote Viability and Nerve Regenerative Ability of Mesenchymal Stem Cells through PDGF-BB/PDGFR-β Signaling. Aging.

[41] Cui Y., Fu S., Hou T., Wu X. (2018). Endothelial Progenitor Cells Enhance the Migration and Osteoclastic Differentiation of Bone Marrow-Derived Macrophages in Vitro and in a Mouse Femur Fracture Model through Talin-1. Cell. Physiol. Biochem..

[42] Pang H., Wu X.-H., Fu S.-L., Luo F., Zhang Z.-H., Hou T.-Y., Li Z.-Q., Chang Z.-Q., Yu B., Xu J.-Z. (2013). Co-Culture with Endothelial Progenitor Cells Promotes Survival, Migration, and Differentiation of Osteoclast Precursors. Biochem. Biophys. Res. Commun..

[43] He T., Peterson T.E., Katusic Z.S. (2005). Paracrine Mitogenic Effect of Human Endothelial Progenitor Cells: Role of Interleukin-8. Am. J. Physiol. Heart Circ. Physiol..

[44] Cheng Q., Lin S., Bi B., Jiang X., Shi H., Fan Y., Lin W., Zhu Y., Yang F. (2018). Bone Marrow-Derived Endothelial Progenitor Cells Are Associated with Bone Mass and Strength. J. Rheumatol..

[45] Kuki S., Imanishi T., Kobayashi K., Matsuo Y., Obana M., Akasaka T. (2006). Hyperglycemia Accelerated Endothelial Progenitor Cell Senescence via the Activation of P38 Mitogen-Activated Protein Kinase. Circ. J..

[46] Zhang Y., Herbert B.-S., Rajashekhar G., Ingram D.A., Yoder M.C., Clauss M., Rehman J. (2009). Premature Senescence of Highly Proliferative Endothelial Progenitor Cells Is Induced by Tumor Necrosis Factor-Alpha via the P38 Mitogen-Activated Protein Kinase Pathway. FASEB J..

[47] Zhao T., Li J., Chen A.F. (2010). MicroRNA-34a Induces Endothelial Progenitor Cell Senescence and Impedes Its Angiogenesis via Suppressing Silent Information Regulator 1. Am. J. Physiol. Endocrinol. Metab..

[48] Song X., Yang B., Qiu F., Jia M., Fu G. (2017). High Glucose and Free Fatty Acids Induce Endothelial Progenitor Cell Senescence via PGC-1α/SIRT1 Signaling Pathway. Cell Biol. Int..

[49] Chen J., Xavier S., Moskowitz-Kassai E., Chen R., Lu C.Y., Sanduski K., Špes A., Turk B., Goligorsky M.S. (2012). Cathepsin Cleavage of Sirtuin 1 in Endothelial Progenitor Cells Mediates Stress-Induced Premature Senescence. Am. J. Pathol..

[50] Kushner E., Van Guilder G., MacEneaney O., Greiner J., Cech J., Stauffer B., Desouza C. (2010). Ageing and Endothelial Progenitor Cell Release of Proangiogenic Cytokines. Age Ageing.

[51] Dykxhoorn D.M., Da Fonseca Ferreira A., Gomez K., Shi J., Zhu S., Zhang L., Wang H., Wei J., Zhang Q., Macon C.J. (2025). MicroRNA-29c-3p and -126a Contribute to the Decreased Angiogenic Potential of Aging Endothelial Progenitor Cells. Int. J. Mol. Sci..

[52] Wang L., Wei J., Da Fonseca Ferreira A., Wang H., Zhang L., Zhang Q., Bellio M.A., Chu X.-M., Khan A., Jayaweera D. (2020). Rejuvenation of Senescent Endothelial Progenitor Cells by Extracellular Vesicles Derived from Mesenchymal Stromal Cells. JACC Basic Transl. Sci..

[53] Kushner E.J., MacEneaney O.J., Weil B.R., Greiner J.J., Stauffer B.L., DeSouza C.A. (2011). Aging Is Associated with a Proapoptotic Endothelial Progenitor Cell Phenotype. J. Vasc. Res..

[54] Zhu S., Deng S., Ma Q., Zhang T., Jia C., Zhuo D., Yang F., Wei J., Wang L., Dykxhoorn D.M. (2013). MicroRNA-10A* and MicroRNA-21 Modulate Endothelial Progenitor Cell Senescence via Suppressing High-Mobility Group A2. Circ. Res..

[55] Zheng Y., Xu Z. (2014). MicroRNA-22 Induces Endothelial Progenitor Cell Senescence by Targeting AKT3. Cell. Physiol. Biochem..

[56] Simoncini S., Chateau A.-L., Robert S., Todorova D., Yzydorzick C., Lacroix R., Ligi I., Louis L., Bachelier R., Simeoni U. (2017). Biogenesis of Pro-Senescent Microparticles by Endothelial Colony Forming Cells from Premature Neonates Is Driven by SIRT1-Dependent Epigenetic Regulation of MKK6. Sci. Rep..

[57] Li X., Fang S., Wang S., Xie Y., Xia Y., Wang P., Hao Z., Xu S., Zhang Y. (2024). Hypoxia Preconditioning of Adipose Stem Cell-Derived Exosomes Loaded in Gelatin Methacryloyl (GelMA) Promote Type H Angiogenesis and Osteoporotic Fracture Repair. J. Nanobiotechnol..

[58] Poulos M.G., Ramalingam P., Gutkin M.C., Llanos P., Gilleran K., Rabbany S.Y., Butler J.M. (2017). Endothelial Transplantation Rejuvenates Aged Hematopoietic Stem Cell Function. J. Clin. Investig..

[59] Ramasamy S.K., Kusumbe A.P., Schiller M., Zeuschner D., Bixel M.G., Milia C., Gamrekelashvili J., Limbourg A., Medvinsky A., Santoro M.M. (2016). Blood Flow Controls Bone Vascular Function and Osteogenesis. Nat. Commun..

[60] Shao H., Xu Q., Wu Q., Ma Q., Salgueiro L., Wang J., Eton D., Webster K.A., Yu H. (2011). Defective CXCR4 Expression in Aged Bone Marrow Cells Impairs Vascular Regeneration. J. Cell. Mol. Med..

[61] Welsh J.A., Goberdhan D.C.I., O’Driscoll L., Buzas E.I., Blenkiron C., Bussolati B., Cai H., Di Vizio D., Driedonks T.A.P., Erdbrügger U. (2024). Minimal Information for Studies of Extracellular Vesicles (MISEV2023): From Basic to Advanced Approaches. J. Extracell. Vesicles.

[62] Wang J., Chen S., Zhang W., Chen Y., Bihl J.C. (2020). Exosomes from miRNA-126-Modified Endothelial Progenitor Cells Alleviate Brain Injury and Promote Functional Recovery after Stroke. CNS Neurosci. Ther..

[63] Wu X., Liu Z., Hu L., Gu W., Zhu L. (2018). Exosomes Derived from Endothelial Progenitor Cells Ameliorate Acute Lung Injury by Transferring miR-126. Exp. Cell Res..

[64] Zhang H., Lu C., Wu L., Li J., Huang M., Tao X., Wu Y., Jia B. (2024). Exosomes Derived from Endothelial Progenitor Cells Ameliorate LPS-Induced Brain Microvascular Endothelial Cells Injury by Delivering miR-126a-5p. Sci. Rep..

[65] Xia J., Song X., Meng J., Lou D. (2022). Endothelial Progenitor Cells-Derived Exosomes Transfer microRNA-30e-5p to Regulate Erastin-Induced Ferroptosis in Human Umbilical Vein Endothelial Cells via the Specificity Protein 1/Adenosine Monophosphate-Activated Protein Kinase Axis. Bioengineered.

[66] Li L., Wang H., Zhang J., Chen X., Zhang Z., Li Q. (2021). Effect of Endothelial Progenitor Cell-Derived Extracellular Vesicles on Endothelial Cell Ferroptosis and Atherosclerotic Vascular Endothelial Injury. Cell Death Discov..

[67] Wu D., Chang X., Tian J., Kang L., Wu Y., Liu J., Wu X., Huang Y., Gao B., Wang H. (2021). Bone Mesenchymal Stem Cells Stimulation by Magnetic Nanoparticles and a Static Magnetic Field: Release of Exosomal miR-1260a Improves Osteogenesis and Angiogenesis. J. Nanobiotechnol..

[68] Lu J., Yang J., Zheng Y., Chen X., Fang S. (2019). Extracellular Vesicles from Endothelial Progenitor Cells Prevent Steroid-Induced Osteoporosis by Suppressing the Ferroptotic Pathway in Mouse Osteoblasts Based on Bioinformatics Evidence. Sci. Rep..

[69] Sun J., Yao C., Luo W., Ge X., Zheng W., Sun C., Zhang Y. (2024). Endothelial Cell-Derived Exosomes Inhibit Osteoblast Apoptosis and Steroid-Induced Necrosis of Femoral Head Progression by Activating the PI3K/Akt/Bcl-2 Pathway. J. Tissue Eng. Regen. Med..

[70] Wang J., Xie X., Li H., Zheng Q., Chen Y., Chen W., Chen Y., He J., Lu Q. (2024). Vascular Endothelial Cells-Derived Exosomes Synergize with Curcumin to Prevent Osteoporosis Development. iScience.

[71] Liu L., Zhou N., Fu S., Wang L., Liu Y., Fu C., Xu F., Guo W., Wu Y., Cheng J. (2024). Endothelial Cell-Derived Exosomes Trigger a Positive Feedback Loop in Osteogenesis-Angiogenesis Coupling via up-Regulating Zinc Finger and BTB Domain Containing 16 in Bone Marrow Mesenchymal Stem Cell. J. Nanobiotechnol..

[72] Cui Y., Fu S., Sun D., Xing J., Hou T., Wu X. (2019). EPC-Derived Exosomes Promote Osteoclastogenesis through LncRNA-MALAT1. J. Cell. Mol. Med..

[73] Wang J., Zhou Z., Chen W., Chen Y., Zheng Q., Chen Y., Ouyang Z., Xu R., Lu Q. (2025). Mechanism of EC-EXOs-Derived THBS3 Targeting CD47 to Regulate BMSCs Differentiation to Ameliorate Bone Loss. Cell Prolif..

[74] Pi Z., Wu Y., Wang X., Li P., Wang R. (2025). Exosomal Manf Originated from Endothelium Regulated Osteoclast Differentiation by Down-Regulating NF-κB Signaling Pathway. J. Orthop. Surg. Res..

[75] Claridge B., Lozano J., Poh Q.H., Greening D.W. (2021). Development of Extracellular Vesicle Therapeutics: Challenges, Considerations, and Opportunities. Front. Cell Dev. Biol..

[76] Gupta D., Wiklander O.P.B., Wood M.J.A., El-Andaloussi S. (2023). Biodistribution of Therapeutic Extracellular Vesicles. Extracell. Vesicles Circ. Nucleic Acids.

[77] Dang X.T.T., Kavishka J.M., Zhang D.X., Pirisinu M., Le M.T.N. (2020). Extracellular Vesicles as an Efficient and Versatile System for Drug Delivery. Cells.

[78] Chen C.W., Wang L.L., Zaman S., Gordon J., Arisi M.F., Venkataraman C.M., Chung J.J., Hung G., Gaffey A.C., Spruce L.A. (2018). Sustained Release of Endothelial Progenitor Cell-Derived Extracellular Vesicles from Shear-Thinning Hydrogels Improves Angiogenesis and Promotes Function after Myocardial Infarction. Cardiovasc. Res..

[79] Zhang Y., Xie Y., Hao Z., Zhou P., Wang P., Fang S., Li L., Xu S., Xia Y. (2021). Umbilical Mesenchymal Stem Cell-Derived Exosome-Encapsulated Hydrogels Accelerate Bone Repair by Enhancing Angiogenesis. ACS Appl. Mater. Interfaces.

[80] Liu Y., Lin S., Xu Z., Wu Y., Wang G., Yang G., Cao L., Chang H., Zhou M., Jiang X. (2024). High-Performance Hydrogel-Encapsulated Engineered Exosomes for Supporting Endoplasmic Reticulum Homeostasis and Boosting Diabetic Bone Regeneration. Adv. Sci..

[81] Wang L., Wang J., Zhou X., Sun J., Zhu B., Duan C., Chen P., Guo X., Zhang T., Guo H. (2020). A New Self-Healing Hydrogel Containing hucMSC-Derived Exosomes Promotes Bone Regeneration. Front. Bioeng. Biotechnol..

[82] Xiang K., Hao M., Zhang Z., Zhang K., Sun H., Zhang L. (2025). Engineering 3D-BMSC Exosome-Based Hydrogels That Collaboratively Regulate Bone Microenvironment and Promote Osteogenesis for Enhanced Cell-Free Bone Regeneration. Mater. Today Bio.

[83] Li X., Si Y., Liang J., Li M., Wang Z., Qin Y., Sun L. (2024). Enhancing Bone Regeneration and Immunomodulation via Gelatin Methacryloyl Hydrogel-Encapsulated Exosomes from Osteogenic Pre-Differentiated Mesenchymal Stem Cells. J. Colloid Interface Sci..

[84] Zhu H., Cheng K., Tian M., Peng Y., Zhang Y., Yan H., Fan S., Shang B., Wu J., Ding H. (2026). 3D-Printed Scaffold Loaded with Baicalin Exosomes Promotes Bone Defect Repair via Mediating PRRX2 to Alleviate Inflammation. iScience.

[85] Zheng W., Zhu Z., Hong J., Wang H., Cui L., Zhai Y., Li J., Wang C., Wang Z., Xu L. (2024). Incorporation of Small Extracellular Vesicles in PEG/HA-Bio-Oss Hydrogel Composite Scaffold for Bone Regeneration. Biomed. Mater..

[86] Li H., Pan H., Feng M. (2026). Enhancing Osteoporosis Treatment: Emerging Roles of Engineered Exosomes in Bone Regeneration and Repair. J. Transl. Med..

[87] Cui Y., Guo Y., Kong L., Shi J., Liu P., Li R., Geng Y., Gao W., Zhang Z., Fu D. (2021). A Bone-Targeted Engineered Exosome Platform Delivering siRNA to Treat Osteoporosis. Bioact. Mater..

[88] Smyth T., Petrova K., Payton N.M., Persaud I., Redzic J.S., Graner M.W., Smith-Jones P., Anchordoquy T.J. (2014). Surface Functionalization of Exosomes Using Click Chemistry. Bioconjugate Chem..

[89] Jay S.M. (2025). Addressing Barriers to Clinical Translation of Extracellular Vesicle Therapeutics. Mol. Ther..

[90] Ma Y., Dong S., Wu A., Jeong S.D., Lee A.S., Jiang W., Kim B.Y.S. (2026). Engineering Challenges and Translational Opportunities in Emerging Gene Delivery Platforms. Nat. Biomed. Eng..

[91] Ma Y., Dong S., Grippin A., Teng L., Lee A.S., Kim B.Y.S., Jiang W. (2025). Engineering Therapeutical Extracellular Vesicles for Clinical Translation. Trends Biotechnol..

[92] Lener T., Gimona M., Aigner L., Börger V., Buzas E., Camussi G., Chaput N., Chatterjee D., Court F.A., Del Portillo H.A. (2015). Applying Extracellular Vesicles Based Therapeutics in Clinical Trials—An ISEV Position Paper. J. Extracell. Vesicles.

[93] Théry C., Witwer K.W., Aikawa E., Alcaraz M.J., Anderson J.D., Andriantsitohaina R., Antoniou A., Arab T., Archer F., Atkin-Smith G.K. (2018). Minimal Information for Studies of Extracellular Vesicles 2018 (MISEV2018): A Position Statement of the International Society for Extracellular Vesicles and Update of the MISEV2014 Guidelines. J. Extracell. Vesicles.

[94] Guzowska J., Kowalski S., Schachta I., Piekuś-Słomka N., Słomka A. (2026). Connecting the Dots: Milestones in the History of Extracellular Vesicle Research. Int. J. Mol. Sci..

[95] Ev-Track Consortium, Van Deun J., Mestdagh P., Agostinis P., Akay Ö., Anand S., Anckaert J., Martinez Z.A., Baetens T., Beghein E. (2017). EV-TRACK: Transparent Reporting and Centralizing Knowledge in Extracellular Vesicle Research. Nat. Methods.

[96] Visan K.S., Lobb R.J., Ham S., Lima L.G., Palma C., Edna C.P.Z., Wu L.-Y., Gowda H., Datta K.K., Hartel G. (2022). Comparative Analysis of Tangential Flow Filtration and Ultracentrifugation, Both Combined with Subsequent Size Exclusion Chromatography, for the Isolation of Small Extracellular Vesicles. J. Extracell. Vesicles.

[97] Busatto S., Vilanilam G., Ticer T., Lin W.-L., Dickson D.W., Shapiro S., Bergese P., Wolfram J. (2018). Tangential Flow Filtration for Highly Efficient Concentration of Extracellular Vesicles from Large Volumes of Fluid. Cells.

[98] Humbert C., Cordier C., Drut I., Hamrick M., Wong J., Bellamy V., Flaire J., Bakshy K., Dingli F., Loew D. (2025). GMP-Compliant Process for the Manufacturing of an Extracellular Vesicles-Enriched Secretome Product Derived from Cardiovascular Progenitor Cells Suitable for a Phase I Clinical Trial. J. Extracell. Vesicles.

[99] Andriolo G., Provasi E., Lo Cicero V., Brambilla A., Soncin S., Torre T., Milano G., Biemmi V., Vassalli G., Turchetto L. (2018). Exosomes from Human Cardiac Progenitor Cells for Therapeutic Applications: Development of a GMP-Grade Manufacturing Method. Front. Physiol..

[100] Mendt M., Kamerkar S., Sugimoto H., McAndrews K.M., Wu C.-C., Gagea M., Yang S., Blanko E.V.R., Peng Q., Ma X. (2018). Generation and Testing of Clinical-Grade Exosomes for Pancreatic Cancer. JCI Insight.

[101] Danilushkina A.A., Emene C.C., Barlev N.A., Gomzikova M.O. (2023). Strategies for Engineering of Extracellular Vesicles. Int. J. Mol. Sci..

[102] Pulido-Escribano V., Torrecillas-Baena B., Camacho-Cardenosa M., Dorado G., Gálvez-Moreno M.Á., Casado-Díaz A. (2022). Role of Hypoxia Preconditioning in Therapeutic Potential of Mesenchymal Stem-Cell-Derived Extracellular Vesicles. World J. Stem Cells.

[103] Li B., Liang A., Zhou Y., Huang Y., Liao C., Zhang X., Gong Q. (2023). Hypoxia Preconditioned DPSC-Derived Exosomes Regulate Angiogenesis via Transferring LOXL2. Exp. Cell Res..

[104] Toghiani R., Azimian Zavareh V., Najafi H., Mirian M., Azarpira N., Abolmaali S.S., Varshosaz J., Tamaddon A.M. (2024). Hypoxia-Preconditioned WJ-MSC Spheroid-Derived Exosomes Delivering miR-210 for Renal Cell Restoration in Hypoxia-Reoxygenation Injury. Stem Cell Res. Ther..

[105] Lei D., Li B., Isa Z., Ma X., Zhang B. (2022). Hypoxia-Elicited Cardiac Microvascular Endothelial Cell-Derived Exosomal miR-210-3p Alleviate Hypoxia/Reoxygenation-Induced Myocardial Cell Injury through Inhibiting Transferrin Receptor 1-Mediated Ferroptosis. Tissue Cell.

[106] Ma S., Zhang Y., Li S., Li A., Li Y., Pei D. (2022). Engineering Exosomes for Bone Defect Repair. Front. Bioeng. Biotechnol..

[107] Yang Z., Shi J., Xie J., Wang Y., Sun J., Liu T., Zhao Y., Zhao X., Wang X., Ma Y. (2020). Large-Scale Generation of Functional mRNA-Encapsulating Exosomes via Cellular Nanoporation. Nat. Biomed. Eng..

[108] Zhao Y., Du L., Han L., Liu F., Chen S., Li Z., Wang F. (2024). Exosomal Hsa_circ_0093884 Derived from Endothelial Progenitor Cells Promotes Therapeutic Neovascularization via miR-145/SIRT1 Pathway. Biomed. Pharmacother..

[109] Pi Z., Wu Y., Wu J., Zhang T., Li P., Wang R. (2025). Endothelial Cell-Secreted Bone Targeting Exosomes Promote Angiogenesis Coupling with Osteogenesis via the PERK-ATF4-CRELD2 Pathway. Stem Cell Res. Ther..

[110] Zha Y., Li Y., Lin T., Chen J., Zhang S., Wang J. (2021). Progenitor Cell-Derived Exosomes Endowed with VEGF Plasmids Enhance Osteogenic Induction and Vascular Remodeling in Large Segmental Bone Defects. Theranostics.

[111] Li F., Wu J., Li D., Hao L., Li Y., Yi D., Yeung K.W.K., Chen D., Lu W.W., Pan H. (2022). Engineering Stem Cells to Produce Exosomes with Enhanced Bone Regeneration Effects: An Alternative Strategy for Gene Therapy. J. Nanobiotechnol..

[112] Li Y., Wang J., Chen S., Wu P., Xu S., Wang C., Shi H., Bihl J. (2020). miR-137 Boosts the Neuroprotective Effect of Endothelial Progenitor Cell-Derived Exosomes in Oxyhemoglobin-Treated SH-SY5Y Cells Partially via COX2/PGE2 Pathway. Stem Cell Res. Ther..

[113] Chaudhari A.P., Budayr O.M., Bonacquisti E.E., Kussatz C.C., Bannon M.S., Law K.J., Liu Y., Bolton M.L., Glassman P.M., Huang L. (2026). The Status of Extracellular Vesicles as Drug Carriers and Therapeutics. Nat. Rev. Bioeng..

[114] Xiong W., Bai X., Zhang X., Lei H., Xiao H., Zhang L., Xiao Y., Yang Q., Zou X. (2023). Endothelial Progenitor-Cell-Derived Exosomes Induced by Astragaloside IV Accelerate Type I Diabetic-Wound Healing via the PI3K/AKT/mTOR Pathway in Rats. Front. Biosci..

[115] Shan S.-K., Lin X., Wu F., Li C.-C., Guo B., Li F.-X.-Z., Zheng M.-H., Wang Y., Xu Q.-S., Lei L.-M. (2024). Vascular Wall Microenvironment: Endothelial Cells Original Exosomes Mediated Melatonin-Suppressed Vascular Calcification and Vascular Ageing in a m6A Methylation Dependent Manner. Bioact. Mater..

[116] Parada N., Romero-Trujillo A., Georges N., Alcayaga-Miranda F. (2021). Camouflage Strategies for Therapeutic Exosomes Evasion from Phagocytosis. J. Adv. Res..

[117] Ben X.-Y., Wang Y.-R., Zheng H.-H., Li D.-X., Ren R., Ni P.-L., Zhang H.-Y., Feng R.-J., Li Y.-Q., Li Q.-F. (2022). Construction of Exosomes That Overexpress CD47 and Evaluation of Their Immune Escape. Front. Bioeng. Biotechnol..

[118] Mao M.-Z., Zheng M.-H., Guo B., Ling Y.-L., Lin X., Li F.-X.-Z., Shan S.-K., Dai D.-X., Qiu L., Cai X.-Y. (2025). FNDC5/Irisin-Enriched sEVs Conjugated with Bone-Targeting Aptamer Alleviate Osteoporosis: A Potential Alternative to Exercise. J. Nanobiotechnol..

[119] Gui L., Ye Q., Yu L., Dou G., Zhou Y., Liu Y., Zhang Y., Yang X., Jin F., Liu S. (2024). Bone-Targeting Peptide and RNF146 Modified Apoptotic Extracellular Vesicles Alleviate Osteoporosis. Int. J. Nanomed..

[120] Lu W., Zeng M., Liu W., Ma T., Fan X., Li H., Wang Y., Wang H., Hu Y., Xie J. (2023). Human Urine-Derived Stem Cell Exosomes Delivered via Injectable GelMA Templated Hydrogel Accelerate Bone Regeneration. Mater. Today Bio.

[121] Jin S., Zhou S. (2026). Exosome-Loaded Hydrogels for Bone Regeneration: A Cell-Free Therapeutic Strategy. J. Biol. Eng..

